# Peptidomics-based identification of antihypertensive and antidiabetic peptides from sheep milk fermented using *Limosilactobacillus fermentum* KGL4 MTCC 25515 with anti-inflammatory activity: *in silico*, *in vitro*, and molecular docking studies

**DOI:** 10.3389/fchem.2024.1389846

**Published:** 2024-04-30

**Authors:** Rinkal Pipaliya, Bethsheba Basaiawmoit, Amar A. Sakure, Ruchika Maurya, Mahendra Bishnoi, Kanthi Kiran Kondepudi, Brij Pal Singh, Souparno Paul, Zhenbin Liu, Preetam Sarkar, Ashish Patel, Subrota Hati

**Affiliations:** ^1^ Department of Dairy Microbiology, SMC College of Dairy Science, Kamdhenu University, Anand, Gujarat, India; ^2^ Department of Rural Development and Agricultural Production, North-Eastern Hill University, Tura Campus, Chasingre, Meghalaya, India; ^3^ Departmentof Agriculture Biotechnology, Anand Agricultural University, Anand, Gujarat, India; ^4^ Regional Center for Biotechnology, Faridabad, Haryana, India; ^5^ Healthy Gut Research Group, Food and Nutritional Biotechnology Division, National Agri-Food Biotechnology Institute, SAS Nagar, Punjab, India; ^6^ Department of Microbiology, School of Interdisciplinary and Applied Sciences, Central University of Haryana, Mahendergarh, India; ^7^ School of Food and Biological Engineering, Shaanxi University of Science and Technology, Xi’an, China; ^8^ Department of Food Process Engineering, National Institute of Technology, Rourkela, India; ^9^ Department of Animal Genetics and Breeding, College of Veterinary Science, Kamdhenu University, Anand, Gujarat, India

**Keywords:** sheep milk, biofunctional properties, peptides, peptidomics, fermentation

## Abstract

This study investigated the synthesis of bioactive peptides from sheep milk through fermentation with *Limosilactobacillus fermentum* KGL4 MTCC 25515 strain and assessed lipase inhibition, ACE inhibition, α-glucosidase inhibition, and α-amylase inhibition activities during the fermentation process. The study observed the highest activities, reaching 74.82%, 70.02%, 72.19%, and 67.08% (lipase inhibition, ACE inhibition, α-glucosidase inhibition, and α-amylase inhibition) after 48 h at 37°C, respectively. Growth optimization experiments revealed that a 2.5% inoculation rate after 48 h of fermentation time resulted in the highest proteolytic activity at 9.88 mg/mL. Additionally, fractions with less than 3 kDa of molecular weight exhibited superior ACE-inhibition and anti-diabetic activities compared to other fractions. Fermentation of sheep milk with KGL4 led to a significant reduction in the excessive production of NO, TNF-α, IL-6, and IL-1β produced in RAW 267.4 cells upon treatment with LPS. Peptides were purified utilizing SDS-PAGE and electrophoresis on 2D gels, identifying a maximum number of proteins bands ranging 10–70 kDa. Peptide sequences were cross-referenced with AHTPDB and BIOPEP databases, confirming potential antihypertensive and antidiabetic properties. Notably, the peptide (GPFPILV) exhibited the highest HPEPDOCK score against both α-amylase and ACE.

## Graphical Abstract

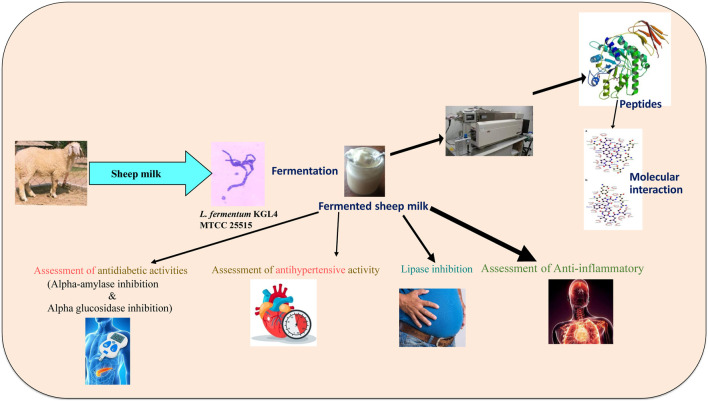

## 1 Introduction

Milk is a favorable substrate for the propagation of lactic acid bacteria (LAB) and the enzymatic breakdown of milk proteins, as indicated by Padhi et al. (2022), leading to the production of numerous bioactive peptides. Throughout the fermentation process, a wide range of microbial-associated proteases, involved in metabolic activities, alter the protein structure in food into peptides (Raveschot et al., 2018). The protein content in milk significantly impacts its nutritional value, and milk proteins and their fractions, especially casein and whey proteins, serve as valuable sources of various bioactive peptides with diverse biological functions. These include antithrombotic, antimicrobial, antioxidant, antihypertensive, and immunomodulatory activities (Park and Nam, 2015).

According to the FAO (2017), sheep milk accounts for approximately 36.5% of total small ruminant milk production globally. There is a rising global demand for sheep milk, and it is estimated that it will have surged by approximately 2.7 metric tonnes (+26%) by 2030 (FAO, 2018). Sheep milk, known for its abundant content of whey protein (1.02–1.3 g/100 g) and casein (4.2–5.2g/100 g) (Selvaggi et al., 2014; Dario et al., 2008) is particularly enriched with these essential components. The consumption of fermented sheep milk is linked to health advantages such as reduced acute inflammation and lowered risk of hypertension and type 2 diabetes. These peptides, derived from a diverse range of fermented foods, play a role in selecting natural and effective ACE inhibitors with minimal or no adverse effects (Baba et al., 2021).

In 2017, 6.7 million deaths globally were attributed to diabetes. Diabetes type 1 impacts over 1.2 million people aged 0–19, with 21 million live births (one in six) experiencing it. As of 26 April 2022, it is projected that 541 million adults will be affected by type 2 diabetes (Lynch, 2022). According to Kannan (2019), India is home to a diabetic population of 77 million, ranking second globally. In managing diabetes, therapeutic interventions target enzymes such as α-amylase, α-glucosidase, and dipeptidyl peptidase-IV (DPP-IV) (Ibrahim et al., 2019). Key enzymes such as α-amylase and α-glucosidase play pivotal roles in breaking down long-chain carbohydrates and releasing absorbable monosaccharides (Sarteshnizi et al., 2021).

Hypertension is a significant contributor to global mortality, leading to a collaborative endeavor to reduce its occurrence by 33% between 2010 and 2030, as outlined in the World Health Organization (WHO) objectives for 2021. In India, the impact of hypertension on the healthcare system is evident from the 2016 Global Burden of Disease (GBD) research, indicating that hypertension alone was responsible for 1.63 million deaths there (Vos et al., 2017). GBD data further disclose that elevated systolic blood pressure plays a substantial role in more than half of the deaths associated with ischemic heart disease (54.2%), chronic kidney disease (54.5%), and stroke (56.2%). With the prevalence of hypertension increasing in India, there is a pressing need to address this health concern (Gupta et al., 2019). The regulation of hypertension involves the renin–angiotensin system (RAS) or renin–angiotensin–aldosterone system, which is essential for controlling peripheral hypertension through ACE-I activity (Baba et al., 2021). Angiotensinogen, continually synthesized by the liver, undergoes transformation from powerful vasoconstrictor angiotensin II (an octapeptide) to angiotensin I (a decapeptide) through the catalytic action of secreted renin in the liver. Additionally, ACE worsens elevated blood pressure by breaking down bradykinin and enkephalins, which function as potent vasodilators (Ningrum et al., 2022).

Changes in contemporary dietary habits and lifestyles have led to an increased susceptibility to disease in humans. In contrast to pharmaceuticals that may have potential side effects, natural foods provide a myriad of health benefits. Small ruminant milk, such as sheep milk, is often required for medicinal purposes, particularly in the case of young children (Haenlein, 2001). The global demand for sheep milk is growing due to its abundant mineral content, elevated levels of protein and fat, and its function as a top supplier of bioactive, functional peptides (BAPs) (Mohapatra et al., 2019). Medical meals can be made with sheep milk, which contains bioactive components. Despite some credible experiences that highlight the medical significance of sheep milk (Razafindrakoto et al., 1993), there is limited research in this domain. However, there is some scholarship that discusses the creation of bioactive peptides (BAPs) from fermented sheep milk utilizing the *Limosilactobacillus fermentum* KGL4 MTCC 25515 strain. This study highlights the ACE-I (angiotensin-converting enzyme inhibition) and antidiabetic properties during the fermentation of sheep milk. The distinctiveness of this research concerns the isolation and identification of peptide sequences displaying ACE-I and antidiabetic effects, employing *Lactobacillus* from the fermented sheep milk of the Panchali breed in Gujarat, India. Additionally, this investigation assesses the potential anti-inflammatory properties of fermented sheep milk using RAW 267.4 cells and also explores the *in silico* analysis of the peptides through a molecular docking study.

## 2 Materials and methods

### 2.1 Culture

The LAB culture *Limosilactobacillus fermentum* KGL4 MTCC 25515 was taken from the Cultural Collection of the Department of Dairy Microbiology, SMC College of Dairy Science, KU, in Anand, Gujarat, India. During the investigation, the preservation and activation of both cultures were carried out using MRS broth media supplied by HiMedia, India, at 37°C.

### 2.2 Sample preparation

The Panchali breed sheep milk was procured from a non-commercial provider in Asodar, Anand, Gujarat, India, under hygienic conditions. The freshly obtained sheep milk underwent filtration using a sanitary muslin cloth followed by prompt heat treatment at 85°C for 10 min, as per Ashokbhai et al. (2022a). Subsequently, the treated milk was preserved in sterile glass containers under refrigerated conditions (5°C ± 1°C) for potential utilization in this research.

To initiate the growth of KGL4 culture, a 2% introduction was performed in sheep milk, and the blend underwent a 24-h incubation at 37°C. Following this, KGL4 was introduced at 2% rates into 10 mL of sterilized, heat-treated sheep milk. Incubation intervals were 0, 12, 24, 36, and 48 h at 37°C. Subsequently, the mixture was centrifuged at 4193 g force for 20 min at 4°C in an Indian-made Plasto Craft centrifuge. The resulting supernatant was then gathered and subjected to filtration with a 0.22-μm Millex®-HV syringe filter (MERK, Ireland). The supernatant was then employed in diverse assays of ACE inhibitory, anti-diabetic (α-glucosidase, lipase, and α-amylase inhibitions), and proteolytic activity.

### 2.3 Assessment of ACE (angiotensin-converting enzyme) inhibition

The percentage inhibition activity of the ACE enzyme using fermented sheep milk supernatants utilizing hippuryl-L-histidyl-L-leucine (HHL) as a substrate was evaluated as per Cushman and Cheung (1971).

### 2.4 ɑ-amylase inhibition activity analysis

The percentage of inhibition activity of the ɑ-amylase enzyme using fermented sheep milk supernatants with 3, 5-dinitrosalicylic acid (DNSA) and soluble starch as a substrate was evaluated (Ademiluyi and Oboh, 2013; Telagari and Hullatti, 2015).

### 2.5 Evaluating the α-glucosidase inhibitory activity

The percentage of inhibition activity of the ɑ-glucosidase enzyme using fermented sheep milk supernatants utilizing 4-nitrophenyl-D-glucopyranoside (P-NPG) as a substrate was determined (Yamaki and Mori, 2006; Shai et al., 2011).

### 2.6 Assessment of lipase inhibition

The percentage of inhibition activity of the lipase enzyme using fermented sheep milk supernatants utilizing 4MUO as a substrate was determined (Kurihara et al., 2003; Sergent et al., 2012).

### 2.7 Assessment of protease activity

The methodology was used to assess the proteolysis using O-phthalaldehyde (OPA). This activity was expressed as mg/mL unit (Hati et al., 2015; Solanki et al., 2017).

### 2.8 Purification, isolation, and characterization of anti-diabetic and ACE-I peptides

The optimization of growth environments involved initiating cultures with a 2.5% inoculation rate and maintaining incubation at 37°C for 48 h. The assessment of peptide content was conducted using the O-phthalaldehyde method (Solanki et al., 2017).

### 2.9 SDS-PAGE evaluation

SDS-PAGE as per Carrasco-Castilla et al. (2012) and LaemmLi (1970) was utilized in order to ascertain the molecular masses of specific protein fragments, following Solanki (2016). The examination involved samples obtained from both unfermented and fermented sheep milk, including the 3 kDa and 10 kDa fractions (permeate and retentate) from fermented sheep milk. A 12% separating gel and 4% stacking gel were employed in the analysis.

### 2.10 2D gel electrophoresis

The refinement process in peptide spots of processed sheep milk water-soluble extracts (WSEs) employed a two-dimensional gel electrophoresis method. This procedure followed Yang et al. (2014), with modifications made to electrical parameters based on the specifications in Panchal et al. (2020).

### 2.11 First dimension-isoelectric focusing (IEF)

A 125-µg protein sample obtained from fermented sheep milk was employed on a ready IPG strip (7 cm) from Bio-Rad, with a pH range of 3–10. Focusing via isoelectric means was conducted with electricity boundaries detailed by Panchal et al. (2020). Following isoelectric focusing, the strip underwent successive immersion in buffer-I equilibrium (10 min) and equilibrium buffer-II (10 min). A 30-s rinse using Bio-Rad’s 1X TGS buffer was then performed before the strip was exposed to SDS-PAGE for two-dimensional (2D) analysis. The gel for the separating phase was prepared as per LaemmLi (1970) and Carrasco-Castilla et al. (2012).

### 2.12 Analysis of peptide fractions through high-performance liquid chromatography in reverse phase (RP-HPLC)

An RP-HPLC system (LC-20 Shimadzu, Japan) for evaluating the synthesis of peptides from fermented sheep’s milk was specifically designed for separating distinct peptide peaks. A Column C18 of UMLSil (United States Thermo Fisher Scientific) featuring an analytical column size of 5 µ, 250 mm × 4.6 mm, white pore was employed in a binary gradient RP-HPLC framework for the separation procedure. A 20-µL micro-injector loop (HAMILTON Bonaduz AG, Switzerland) was utilized to inject the specimen. Eluent: A was composed of TFA at 0.01% (v/v) within deionized water, while Eluent: B had an 80:20 ratio deionized water to acetonitrile with the same TFA content. At room temperature, the separation was performed at a 0.25 mL/min flow rate. The peptides were discharged in gradient manner: 0–1 min, 10% B; 1–10 min, 20% B; 10–15 min, 25% B; 15–20 min, 35% B; 20–30 min, 50% B; 30–33 min, 60% B; 33–36 min, 70% B; 36–39 min, 80% B; 39–50 min, and 90%–100% B. The absorption was detected using a Shimadzu SPD-20A UV/Visible Wavelength Detector set at 214 nm. The integration of all peaks facilitated the calculation of the overall peak area (Parmar, 2017).

### 2.13 Production of peptides using high-performance liquid chromatography in the reverse phase

Peptides were separated from ultra-filtered fractions using the RP-HPLC method obtained from fermented sheep milk with KGL4 *Lactobacillus*. Inoculated at a 2.5% rate, KGL4 was centrifuged for 30 min at 4193 g force after being incubated for 48 h at 37°C. After centrifugation, an extract of water-soluble fermented sheep milk was isolated and underwent ultra-filtration employing membranes with two different molecular weight cut-offs (3 kDa and 10 kDa). Thus resulted in retentate and permeate fractions and was further analyzed for ACE inhibition (2.3) and antidiabetic assays, including lipase inhibition (2.5), α-amylase (2.4), and α-glucosidase inhibition (2.5)

### 2.14 Identification and characterization for separated peptides using RPLC/MS

#### 2.14.1 Liquid chromatography

Liquid chromatography was carried out with an Ad hoc column (2.1 mm × 100 mm, 1.5 μm, India) with a sample temperature of 20°C and a column temperature of 40°C. The mobile phases A and B were acetonitrile (ACN) with 0.1% formic acid and water, respectively. In 2D gel, protein spots were isolated and underwent digestion by in-gel trypsin. The resulting peptides that were digested by trypsin were passed through a nylon filter measuring 0.22 µm before injection in 20 µL capacity flowing at 0.3 mL/min. The gradient column elution parameters were optimized based on Parmar (2017), and the procedure was then carried out.

The ABSCIEX QTRAP 4500 and Ekspert ultra LC 100 (Eksigent, United States), together with electron spray ionisation (ESI) interaction, were used for the mass spectrometry study. Recognizing unidentified peptides in fermented sheep milk was accomplished through reverse-phase liquid chromatography mass spectrometry (RPLC-MS), switching from enhanced product ion (EPI) to enhanced mass spectra for scanning. A tailored method was developed for identifying masses within the 350–2,000 dalton range in EMS scan mode, with specific parameters such as a delustering potential (DP) of 80, an electron potential (EP) of 10, and a core energy (CE) of 5500 ESI volts using a rolling-circle energy-ramping function. The EPI scan was configured to identify ions in the range of 100–2,000 daltons employing high collision-induced dissociation (CAD) and maintaining consistent DP and EP values. Criteria for information-dependent acquisition (IDA), involving tracking the top one to three peaks while maintaining a 250 mDa mass tolerance, were applied for mass scanning. Additionally, an isotopic pattern utilizing an enhanced resolution scan detected objects of different masses.

#### 2.14.2 Analysis of peptide identification

The mass spectra produced by LC‒MS were examined utilizing ProteinPilot software to identify peptide sequences. These sequences were subsequently compared with the Anti-Hypertensive Peptides Database (AHTPDB) to confirm ACE inhibitory activity and with BIOPEP to validate antidiabetic activity.

### 2.15 Anti-inflammatory effects of fermented sheep milk

#### 2.15.1 Cell culture

Cells were procured from the Centre for National Cell Science in Pune, India. DMEM (Dulbecco’s modified Eagle medium) and PBS (phosphate-buffered saline) were sourced through Lonza Bioscience in Switzerland, and P/S (penicillin/streptomycin) was acquired from Gibco, Thermofisher Scientific, in the USA. MP Biomedicals supplied the fetal bovine serum (FBS), and Cusabio Biotech in China provided the lipopolysaccharide (LPS). The ELISA kit for TNF-α, IL-6, and IL-1β was purchased via Elabscience in the United States.

#### 2.15.2 Cell viability

The 264.7 RAW cells were subjected to subculture following a 48-h incubation with DMEM enhanced with 1% P/S and 10% FBS (fetal bovine serum) as per Khare et al. (2020). In accordance with this protocol, 96-well plates were employed, each containing 1 × 10^5^ cells, and placed in a humid incubator at 37°C with 5% CO_2_ for 16 h. Afterward, the cells were exposed to fermented sheep milk (KGL4) at concentrations of 2, 1, and 0.5 mg/mL for 24 h. The MTT assay was then conducted by including 3-(4, 5-dimethylthiazol-2-yl)-2,5-diphenyltetrazolium bromide @ 0.5 mg/mL. Cells had been kept in darkness for 4 hours with 5% CO_2_ at 37°C. Formazan crystals were dissolved by adding 0.1 mL of DMSO (dimethyl sulfoxide), and a Tecan Life Science M200 PRO microplate reader was employed to detect at 570 nm.

#### 2.15.3 Production of nitric oxide (NO)

The 264 RAW.7 cells were cultivated for 24 h in accordance with protocols after being deposited in a 48-well plate at a density of 2 × 10^5^ cells per well. Subsequently, macrophages that were fully confluent were subjected to 1 μg/mL lipopolysaccharide (LPS) in combination with three specific *Lactobacillus* strains (KGL4) used for fermenting sheep milk. The concentrations of these *Lactobacillus* cultures were 2, 1, and 0.5 mg/mL. After this treatment, the cells were kept in a humidified CO_2_ incubator for 16 h. Using the Griess reagent, nitrite levels were ascertained, and absorption of optical density at 540 nm was noted. The supernatants that were extracted from the cultures were subjected to cytokine analysis.

#### 2.15.4 IL-6, TNF-α, and IL-1β cytokine measurements

TNF-α, IL-6, and IL-1β levels in the supernatants from cell cultures were quantified using of a commercial ELISA kit as per the manufacturer’s instructions (Elabscience, United States).

### 2.16 Molecular docking

The methodology for the molecular docking of a specific peptide adhered to the procedure outlined by Singh et al. (2024). In summary, the peptide’s 3D structure was anticipated by employing the AlphaFold Colab tool by Google Deep Mind (Mirdita et al., 2022). The three-dimensional structures of pancreatic α-amylase (1DHK) and angiotensin-converting enzyme (1O86) were obtained from the RCSB PDB database. The protein-active sites were located using the Active Site Prediction server Singh et al. (2011). The chosen peptide served as the ligand, and the respective enzymes were identified as receptors for the Vina AutoDock v1.2.0 docking procedure (Trott and Olson, 2010). Additionally, the docking score for the peptide was predicted via HPEPDOCK 2.0, which was adaptable; blind peptide docking entails rapidly modeling amino acid configurations and then worldwide binding orientation sampling (Zhou et al., 2018).

### 2.17 Statistical analysis

Analysis was executed using a CRD (completely randomized design) as outlined by Steel and Torrie (1980). The importance of every parameter’s impact on the particular trait was evaluated @ a 5.0% significance level. Software, Inc. GraphPad Prism 8.0 (United States: La Jolla, CA) was utilized for data analysis. One-way ANOVA was used for multiple group comparisons and then *post hoc* analysis using Tukey. Statistical significance was considered at *p* ≤ 0.05. The entire set of experiments was replicated thrice.

## 3 Results and discussion

### 3.1 ACE-inhibitory activity of fermented sheep milk

**FIGURE 1:**
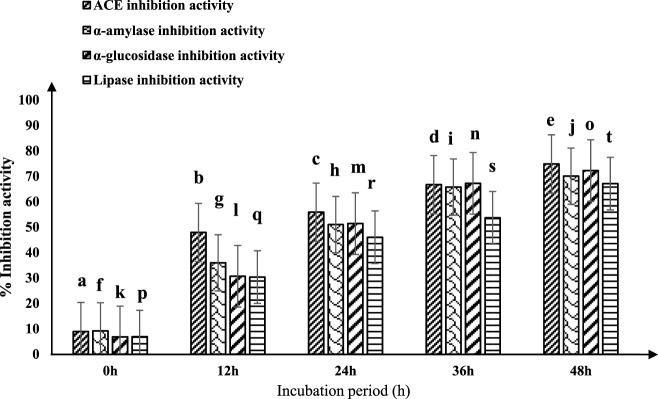
ACE-inhibitory and antidiabetic activities of fermented sheep milk using KGL4 *ACE-inhibitory and antidiabetic activity (%) mean ± SD of three replicate studies (*n* = 3); values with different superscripts differ significantly (*p* < 0.05).

The ACE-inhibitory activity of fermented sheep milk utilizing the *Limosilactobacillus fermentum* (KGL4) culture is shown in Figure 1. A significant increase in ACE inhibition activity (*p* < 0.05) was noted throughout the incubation period. The fermented sheep milk demonstrated ACE-inhibitory effects ranging from 8.91% ± 2.33% to 74.82% ± 1.2% within the 0–48-h time frame at 37°C (Figure 1). The highest ACE inhibitory activity, reaching 74.82% ± 1.2%, was noticed following a 48-h incubation period at 37°C, surpassing the inhibition levels recorded at other incubation times.

The ACE inhibition (ACE-I) efficacy of fermented sheep milk displayed significant variability across different incubation durations, indicating a notable rise in ACE enzyme inhibition with prolonged incubation times (*p* < 0.05). This trend aligns with observations by Solanki et al. (2017). Fermented milk generated by diverse *Lactobacillus* species is known for its substantial ACE-I activity exceeding 50% (Hati et al., 2018; Wu et al., 2019). Changes in ACE-I activity observed between lactic acid bacteria are linked to their protease function in fermented milk, and specific components of lactic acid bacteria proteolysis play a role in influencing this activity (Chen et al., 2015).

Mudgil et al. (2023a) explored the inhibitory activity of the angiotensin-converting enzyme (ACE-I) of sheep milk which was fermented with five probiotic bacteria under refrigerated conditions for 0 , 7 , and 14 days. The ACE-IC50 values for sheep milk fermented with various probiotic microorganisms—*L. fermentum*, *L. pentosus*, *L. plantarum* ssp. *Argentoratensis*, *E. hirae*, and *P. pentosaceus*—were recorded, respectively, as 842.0, 1079, 1079, 1060.5, and 2071.4 µg protein equivalent per milliliter at 0 days, 668.3, 703.0, 659.8, 659.8, and 905.3 µg protein equivalent per milliliter after 7 days, and 301.0, 445.4, 430.6, 455.8, and 445.0 µg protein equivalent/mL after 14 days of storage. The ACE-IC_50_ values observed after 24 h, 7 days, and 14 days of fermentation ranged from 842.0 to 2071.4, 842.0 to 2071.4, and 659.8 to 905.3 protein equivalent/mL, respectively, suggesting a substantial increase in ACE inhibitory potential with prolonged incubation. This research supports our findings and also highlights a consistent upward trajectory in ACE-I activity with extended fermentation periods. Balthazar et al. (2019) conducted trials using semi-skimmed sheep milk blended with strawberries, contrasting it with yogurt starter cultures that included *Lactobacillus plantarum* strain (CECT 8328) possessing promising probiotic characteristics. They also integrated commercial prebiotic components into five distinct formulations—SMB1: yogurt-flavored drink; SMB2: possible beverage with probiotic culture; SMB3: a possible beverage with probiotic culture and inulin; SMB4: a potato starch beverage with a possible probiotic bacterium; SMB5: possible probiotic beverage made from potato starch cultures. These outcomes revealed an augmentation in the antihypertensive efficacy of all five prebiotic-enriched products during the initial 0–30 days of storage, with SMB5 demonstrating the most substantial antihypertensive effect (up to 60%). Balthazar et al. (2019) concluded that the combination of probiotics and prebiotics in sheep milk showcased ACE-I activity (60%). Nonetheless, our investigation proposes that the KGL4 culture, when used independently in fermentation, displayed a better capacity for peptide release than these observations.

### 3.2 Fermented sheep milk anti-diabetic activity

The efficacy of fermented sheep milk in producing antidiabetic effects is shown in Figure 1. Significant variations (*p* < 0.05) in antidiabetic outcomes were observed during the incubation period within the 0–48-h interval. The inhibitory actions against crucial enzymes associated with diabetes—α-amylase, α-glucosidase, and lipase—ranged from 9.16% ± 2.39% to 70.02% ± 1.48%, 6.77% ± 1.88% to 72.19% ± 0.42%, and 6.90% ± 1.93% to 67.08% ± 1.05%, respectively. Following 48 h of incubation at 37°C, fermented sheep milk showcased its most potent antidiabetic effects, achieving a 70.02% inhibition of alpha-amylase, 72.19% inhibition of alpha-glucosidase, and 67.08% inhibition of lipase. Larosa et al. (2021) observed extended incubation periods to enhance the potential antidiabetic effects of sheep milk kefir. Their research involved preparing six kefir formulations from sheep milk, incorporating different sugars—Brown (KIII), fructose (KIV), sucrose (KI), demerara (KII), coconut sugar (KV), and honey (KVI). This formulation underwent a 28-day fermentation process using a combination of *Lactococcus lactis* spp. *lactis* biovar *diacetyl lactis*, *Lactobacillus brevis*, *Lactococcus lactis* ssp. *lactis, Leuconostoc* spp., and *Saccharomyces bayanus*. The primary focus of the investigation centered on evaluating the α-glucosidase, α-amylase, and ACE-inhibitory activity in the samples of sheep milk kefir. Their findings indicated variations in ACE inhibitory activity (11.6%–49.2%), α-amylase inhibition (19.3%–56.7%), and α-glucosidase inhibition (24.3%–39.3%). Notably, incorporating non-traditional sugars (KII-KVI) improved the functional characteristics of fermented milk. This led to a noteworthy rise in α-amylase inhibition (18%–37.4%) and ACE inhibitory action (27.5%–37.6%) (*p* < 0.05) in contrast to the mixture containing sucrose. The most significant enhancements in functional properties were observed in kefir formulations containing honey (KVI) and coconut sugar (KV) for the ACE-inhibitory properties (*p* < 0.05), as well as kefir with fructose (KIV), brown sweets (KIII), and demerara sweets (KII) to inhibit α-amylase (*p* < 0.05). Furthermore, only KIII, KIV, and KV formulations demonstrated a noteworthy increase in α-glucosidase inhibition (*p* < 0.05). Balthazar et al. (2019) investigated fermented sheep milk and strawberry beverages utilizing *Lactobacillus plantarum* strain (CECT 8328), known for its possible probiotic qualities. The inhibitory effects of α-glucosidase and α-amylase at both 0 and 30 days of storage were assessed. The results revealed a significant increase of around 7% and 8%, respectively, in inhibition after 30 days compared to the initial measurements at 0 days. Among the formulations, SMB5, recognized as a potential probiotic culture potato starch beverage, demonstrated the highest α-amylase inhibition (>65%), while SMB4 exhibited more effective α-glucosidase inhibition at 36.13%. These findings are consistent with our own results, providing support for the observed improvement in the inhibition of α-glucosidase and α-amylase with extended incubation time.

Khakhariya et al. (2023) investigated the potential antidiabetic consequences of fermenting camel and buffalo milk using *Lacticaseibacillus paracasei*, MG027695 (M11) during incubation periods ranging from 12 to 48 h. The inhibition of α-glucosidase, α-amylase, and lipase with fermented buffalo milk varied 33.09%–59.95%, 57.09%–79.14%, and 40.75%–63.71%, respectively. Similarly, fermented camel milk exhibited inhibitory activities (inhibition of α-glucosidase, α-amylase, and lipase) of 46.27%–64.55%, 61.43%–81.66%, and 50.15%–69.83%, respectively. Notably, after incubation at 37°C for 48 h, buffalo and camel milk both demonstrated very robust effects against diabetes. Kinariwala et al. (2020) studied the fermented milk combined alongside *L. fermentum* (M2) and *L. fermentum* (M7) for 24 h at 37°C, reporting lipase inhibitory activity of 24.24% for M2 and 18.99% for M7. While their observations were made at 24 h, our study extended the incubation period to 48 h, resulting in higher lipase inhibition activity due to the prolonged incubation times with strong proteolytic activity. This is consistent with the observed increase in α-amylase, α-glucosidase, and lipase inhibitory action with longer incubation.

### 3.3 Optimizing fermented sheep milk for the production of peptides

**FIGURE 2:**
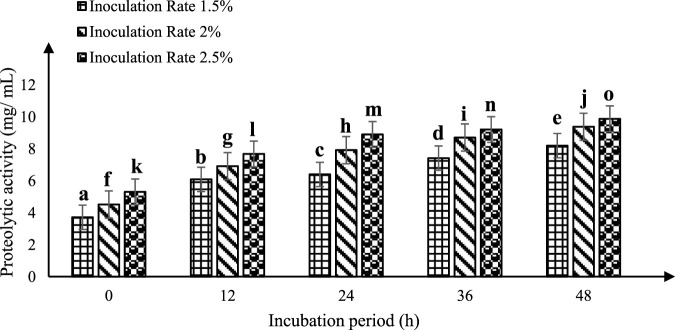
Proteolytic activity (mg/mL) of fermented sheep milk using KGL4 *Mean ± SD of three replicates; each sample was subjected to three replicates; values with different superscripts differ significantly (*p* < 0.05).

The evaluation of enzymatic activity was carried out using O-phthalaldehyde (OPA) as a substrate in a spectrophotometric assay at 340 nm, as per Solanki et al. (2017). To optimize peptide production while sheep’s milk is fermenting, a KGL4 strain culture of *Lactobacillus* was employed. Different inoculation amounts (1.5%, 2.0%, and 2.5% v/v) of this specific strain were introduced into heat-treated milk from sheep and then cultivated for 0, 12, 24, 36, and 48 h at 37°C. Notable variations (*p* < 0.05) within proteolytic (OPA) activity were noted with respect to both incubation time and inoculation rate. The enzymatic activity demonstrated an increase with prolonged incubation periods at each inoculation rate (Figure 2). Maximum proteolytic (OPA) activity was recorded following a 2.5% inoculation rate for 48 h of incubation (9.88 ± 0.11 mg/mL), exceeding the rates of 1.5% (8.20 ± 0.34 mg/mL) and 2.0% (9.38 ± 0.27 mg/mL).

Ashokbhai et al. (2022a) explored the proteolytic capabilities of sheep milk using the OPA method. Additionally, they investigated the influence of fermenting sheep milk with *Lactiplantibacillus plantarum* (KGL3A) across different conditions, including incubation periods of 12, 24, 36, and 48 h, and rates of 1.5%, 2%, and 2.5% culture inoculations. The observed action of proteolysis ranged from 6.10 mg/mL (at 1.5% inoculation following 12-h incubation time) to 10.40 mg/mL (at 2.5% inoculation following 48-h incubation time ). Their findings revealed a significant augmentation in proteolytic activity as the incubation time prolonged (*p* < 0.05). Additionally, the level of proteolytic activity demonstrated variability based on the inoculation rate (1.5%, 2.0%, and 2.5%). Importantly, a noteworthy increase in proteolytic activity was noted after 48 h for all inoculation rates, compared to the 12-, 24-, and 36-h incubation periods. Parmar et al. (2020) investigated the proteolytic capabilities of two strains of *Lactobacillus*—*L. fermentum* (TDS030603) (MTCC 25067) (LF) and *L casei* (KR732325) (NK9)—in the context of Surti Indian breed goat milk (*Capra aegagrus hircus*). They observed a significant enhancement (*p* < 0.05) in the proteolytic activity of LF and NK9 in goat milk across various incubation durations (0, 6, 12, 24, and 48 h) and various rates of inoculation (1.0, 1.5, and 2.0%). Remarkably, both LAB cultures—LF (9.709 mg/mL) and NK9 (7.598 mg/mL) —exhibited substantial proteolytic effects after a 48-h incubation period. Our findings align with these results, highlighting that proteolytic activity is positively influenced by both incubation time and inoculation rate. Our investigation specifically revealed the maximum activity of proteolysis at 48 h, employing a rate of 2.5% inoculation. Moreover, the action of proteases of KGL4 also demonstrated a significant increase (*p* < 0.05) with an extended incubation times (0, 12, 24, 36, and 48 h), reaching its peak (9.88 ± 0.11 mg/mL) at 48 h with a 2.5% inoculum rate. Collectively, these studies affirm the consistent trend that proteolytic activity tends to rise with extended incubation time and is highest at an inoculation rate of 2.5% following a 48-h incubation period.

### 3.4 Assessment of the ACE-inhibitory and antidiabetic qualities of ultra-filtered WSE fractions from fermented sheep milk using RP-HPLC analysis

**FIGURE 3:**
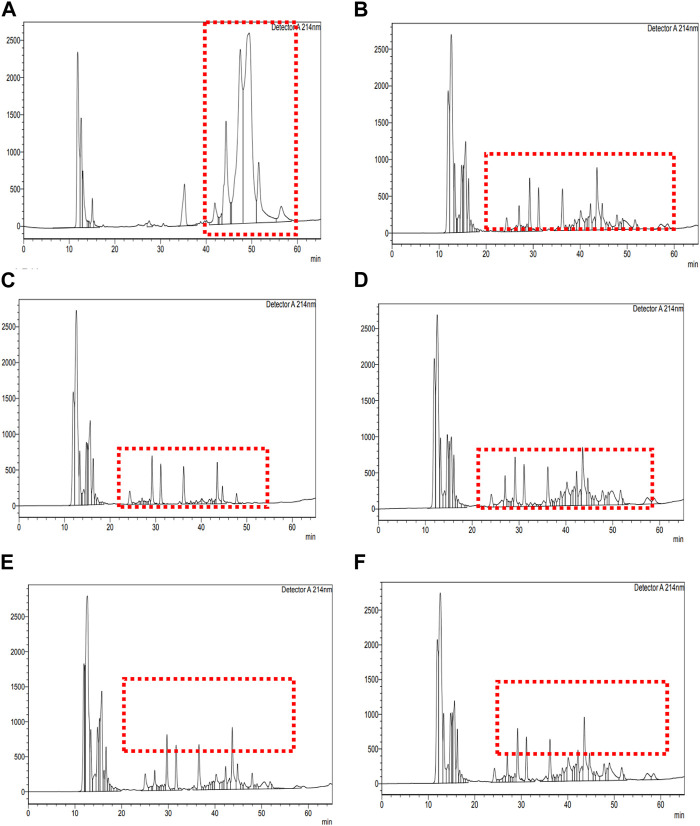
RP-HPLC chromatogram of unfermented sheep milk. RP-HPLC chromatograms of fermented sheep milk with KGL4 culture and fractionated components: **(A)** unfermented sheep milk; **(B)** fermented sheep milk using KGL4 culture; **(C)** 3 kDa permeate from fermented sheep milk using KGL4 culture; **(D)** 3 kDa retentate from fermented sheep milk using KGL4 culture; **(E)** 10 kDa permeate from fermented sheep milk using KGL4 culture; **(F)** 10 kDa retentate from fermented sheep milk using KGL4 culture.

The present study included examining retentate and permeate samples resulting from the ultrafiltration of peptides derived from fermented sheep milk, employing molecular weight cut-offs of 3- and 10-kDa membranes. Figure 3a of unfermented sheep milk and Figures 3b–f of fermented sheep milk display RP-HPLC chromatograms obtained under optimized growth conditions (2.5% inoculation rate, up to 48 h, at 37°C). Table 1 presents a characterization of peptide fractions (< 3 kDa, > 3 kDa, < 10 kDa, > 10 kDa) derived from sheep milk post-fermentation, utilizing RP-HPLC.

**TABLE 1:** Characterization of <3 kDa, >3 kDa, <10 kDa, and >10 kDa peptides generated from fermented sheep milk measured using RP-HPLC.

Culture	Sample (kDa)	Number of peaks	Retention time range (min.)
KGL4	<3	30	24.33 to 48.53
	>3	44	24.08 to 58.86
	<10	44	25.04 to 57.52
	>10	40	24.22 to 58.47

**FIGURE 4:**
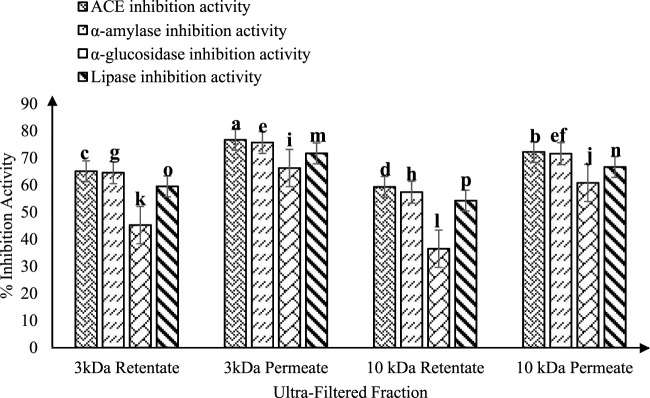
ACE-inhibitory and antidiabetic activities of ultra-filtered fractions (3 and 10 kDa permeate and retentate) from fermented sheep milk (KGL4). *Values with different superscripts differ significantly (*p* < 0.05), ACE-inhibitory and antidiabetic activities (%) Mean ± SD of three replicate experiments (*n* = 3).

Figure 3A illustrates the RP-HPLC chromatogram of non-fermented sheep milk, indicating a significant increase in peptide production in fermented samples compared to their non-fermented state. The KGL4 peaks, found in the 3–10 kDa range, especially displayed the highest number of peaks, with retention times of 24.08–57.52 min (Table 1). Non-fermented milk contained intact proteins, while fermented milk contained diverse protein fragments due to the strong proteolytic action of the KGL4 strain. Some fragments were also found in the control, possibly due to heating or inherent proteolytic enzymes in sheep milk. The fermented sheep milk’s 3 kDa permeate sample displayed the highest levels of hypoglycemic and ACE-inhibiting activities in contrast to membranes with different cut-off values. Specifically, the 3-kDa permeate exhibited optimal ACE-inhibitory (76.69% ± 2.11%), α-amylase inhibitory (75.69% ± 3.49%), α-glucosidase inhibitory (66.27% ± 2.69%), and lipase inhibitory (71.67% ± 1.75%) activities (Figure 4). ACE-I peptides, typically with a weight below 3 kDa and ranging from 6 to 16 amino acids, play a vital part. Furthermore, a peptide's effectiveness in addressing diabetes is influenced by its alanine sequence and place in the peptide order. Amino acids with a molecular weight below 3 kDa—including proline, phenylalanine, leucine, and glycine—exhibited sharp α-amylase inhibition, possibly due to the inhibitory effects of these specific amino acid residues. Additionally, Mu et al. (2023) emphasized that peptides containing two to eight amino acid compounds, particularly those with more amino acids that are hydrophobic such as proline and leucine, demonstrated increased inhibitory effects on α-glucosidase.

According to Rubak et al. (2022), ACE inhibitory effects are observed in skimmed goat milk (11% w/v) fermented with *L. lactis* ssp. *lactis* BD17 (2% v/v) for up to 48 h. The study found significant ACE inhibition in the supernatant—portions of the filtered fermented milk that are >3 kDa and <3 kDa. The ACE inhibition percentages for the supernatant and the >3 kDa and <3 kDa fractions were 60.33%, 24.57%, and 57.31%, respectively. In particular, no statistically significant difference was observed (*p* > 0.05) within ACE-I action among the portion of 3 kDa and the aqueous phase. The research also identified an ACE-I peptide with a molecular weight of approximately 657–1,994 Da/2 kDa and composed of six–eighteen peptides. Parmar et al. (2018) investigated fermented goat milk using *L. fermentum* TDS030603 (LF) (MTCC 25067) and *L. casei* KR732325 (NK9). They utilized centrifugation and ultrafiltration through membranes of 3, 5, and 10 kDa to obtain retentate and permeate samples. The ACE-I activity percentages for NK9 were 36.26%, 22.00%, and 25.16% in the 3 kDa, 5 kDa, and 10 kDa permeate samples, respectively, while LF exhibited 14.40%, 22.28%, and 31.49% ACE-I activity, respectively. In the retentates of 3, 5, and 10 kDa, NK9 showed ACE-I action of 43.58%, 30.88%, and 33.19%, and LF demonstrated 29.46%, 33.62%, and 37.77% ACE-I activity, respectively. These results suggest that a molecular weight cut-off of 3 or 5 kDa could be deemed appropriate. Remarkably, their study substantiates our findings, indicating that the most potent ACE-I peptides are present in the molecular weight fraction below 3 kDa. Shirkhan et al. (2023) examined the effects of LAB strains (*L. paracasei* ssp. *paracasei*, *L. delbrueckii*, and *L. helveticus*) on UHT milk. These LAB strains were observed in order to diminish the efficacy of α-amylase and α-glucosidase enzymes in milk that was fermented and treated with fractions of 3 kDa, 5–10 kDa, and >10 kDa. While all fractions from *L. helveticus* displayed inhibiting impacts on α-glucosidase and α-amylase enzymes, certain peptide fractions from different strains did not demonstrate enzyme inhibition. The assessment of the antidiabetic potential of bioactive peptides relied on the IER (inhibitory efficiency ratio), suggesting that the lowest molecular weight peptide portions exhibited the most pronounced hypoglycemic effects. Shirkhan et al. (2023) underscored the noteworthy impact of fraction size on enzyme inhibition (*p* < 0.05) with the >10 kDa peptide fraction. Lower molecular-weight peptide fractions showed increased proteolysis and antidiabetic properties and displayed the lowest inhibition rate for both enzymes. Lower molecular-weight peptide fractions showed increased proteolysis and antidiabetic properties (*p* < 0.05). These findings corroborate the current results and suggest that anti-diabetic effects are more potent in low molecular-weight peptide fractions than in higher molecular-weight proteins.

### 3.5 Analysis of SDS-PAGE from fermented sheep milk

**FIGURE 5:**
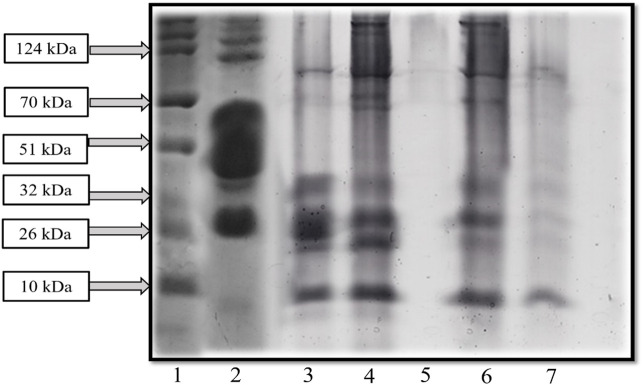
SDS-PAGE exhibited the protein profile of fermented sheep milk with KGL4 culture (1: protein ladder, 2: unfermented sheep milk, 3: fermented sheep milk, 4: 3 kDa retentate, 5: 3 kDa permeate, 6: 10 kDa retentate, and 7: 10 kDa permeate).

SDS-PAGE analysis was conducted on whole sheep-milk extracts cultured and fermented using the KGL4 strain. A low molecular-weight ladder ranging from 10 to 315 kDa was utilized. A comparison of SDS-PAGE profiles between unfermented and fermented sheep’s milk revealed an increased number of protein bands in the latter, attributed to the LAB’s robust proteolytic activity. Figure 5 illustrates that sheep milk without fermentation exhibited bands of protein of 26–315 kDa in the SDS-PAGE. However, sheep milk fermented with KGL4 displayed bands of 10–32 kDa and 51–70 kDa regions. Ashokbhai et al. (2022b) explored *Lactobacillus plantarum* (KGL3A) strains obtained from fermented and unfermented sheep milk through SDS‒PAGE analysis. Their results revealed that the unfermented sheep milk displayed larger protein bands than the fermented milk. The lactic acid bacteria action resulted in the breakdown of these protein bands into smaller peptides. In unfermented sheep milk, the bands of protein ranged between 10 and 42 kDa while, in fermented sheep milk, the bands had a length of 10–70 kDa. Sansi et al. (2023) utilized SDS‒PAGE to analyse goat milk casein and whey protein. They identified major whey proteins such as lactoferrin, serum albumin, immunoglobulin (IgG), α-lactalbumin (around 14 kDa), and β-lactoglobulin (ranging from 14 to 25 kDa) as associated with specific bands. Additionally, in bands of 25–75 kDa, the three main proteins in casein were found to be α, β, and κ-casein. However, our study observed protein bands ranging from less than 10 kDa to 124 kDa in length. Parmar et al. (2020) investigated the properties of whey protein concentrates from goat milk subjected to fermentation using *L. fermentum* (M5) (KU366365) and *L. paracasei* (M16) (KU366368). Compared to unfermented goat milk, SDS‒PAGE analysis indicated a decline in protein bands in fermented goat milk, attributed to the lactic acid bacteria’s proteolytic activities. Unfermented goat milk displayed bands of protein 75‒11 kDa. Both samples of fermented goat milk (M5 and M16) as well as goat milk without fermentation exhibited peptides of low molecular weight of about 25 kDa, with only M16 containing 11 kDa. The study’s findings supported the conclusion that lactic fermentation resulted in reduced native protein size due to protein breakdown.

### 3.6 Two-dimensional gel electrophoresis of fermented sheep milk

**FIGURE 6:**
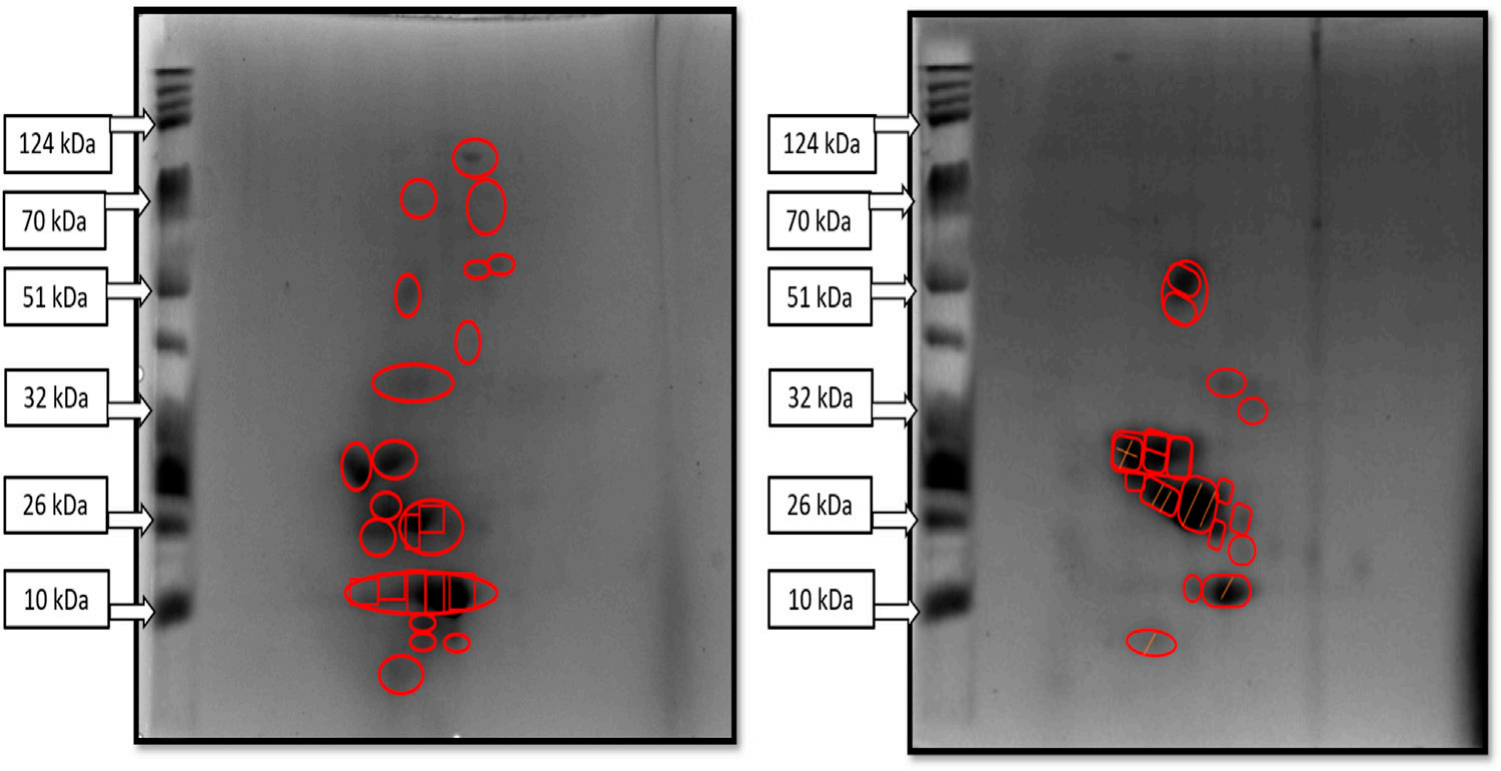
2D gel electrophoretic image of sheep milk fermented with KGL4 culture. Two images showing different spots of purified protein samples in 2D gel images obtained from fermented sheep milk with KGL4 culture; these spots were injected in LCMS for peptide sequencing after in-gel trypsin digestion.

Two-dimensional (2D) electrophoresis separation of proteins in fermented sheep milk employed an IPG strip with a pH range of 3–10, according to molecular weight and isoelectric point. In the examination of the KGL4 strain fermented with sheep milk, 36 unique spots were identified on the 2D gel electrophoresis. The fragments of protein produced by the KGL4 strain displayed a molecular weight distribution between 10 and 51 kDa, with the concentration of spots primarily falling within 10 kDa–32 kDa (Figure 6). The patterns observed in the figures suggest the occurrence of protein degradation and the presence of smaller molecular weight peptides during the lactic acid bacteria-mediated fermentation of sheep milk. Ashokbhai et al. (2022b) conducted a study that evaluated the protein composition of sheep milk fermented with *Lactobacillus plantarum* (KGL3A). Utilizing 2D gel electrophoresis, they identified 13 protein spots ranging in size from 10 to 70 kDa. The results indicated a reduction in protein size post-fermentation, attributed to proteolysis, with the observed spots predominantly falling within the smaller molecular weight range. This trend was consistent across all three cultures investigated in their study. Panchal et al. (2020) identified 39 spots in 2D electrophoresis on gels for *Lactobacillus fermentum* (M4) fermented proteins from goat milk. In our investigation, all three *Lactobacillus* cultures exhibited the maximum number of spots within the atomic molecular weight range of 10–32 kDa. Shukla et al. (2022) used *L. plantarum* KGL3A to investigate the amino acid content of fermented camel milk, with gel electrophoresis (2D) revealing 31 spots with atomic weights of 10 to 100 kDa, attributed to the proteolytic action of *L. plantarum*. Notably, the protein spots generated by KGL4 and M11 ranged from 10 to 51 kDa, while KGL3A exhibited a broader spectrum of 10–70 kDa. In their study on kefir proteomics, Rubak et al. (2020) employed 2D electrophoresis and identified protein spots ranging from 14 to 97 kDa, with a predominant presence in the 14–31 kDa range. Both investigations substantiate our findings, supporting the inference that the fermentation by lactic bacteria that produces lactic acid results in the formation of smaller molecular-weight proteins from milk protein.

### 3.7 Identification of purified peptides and characterisation employing RP-LC/MS

**FIGURE 7:**
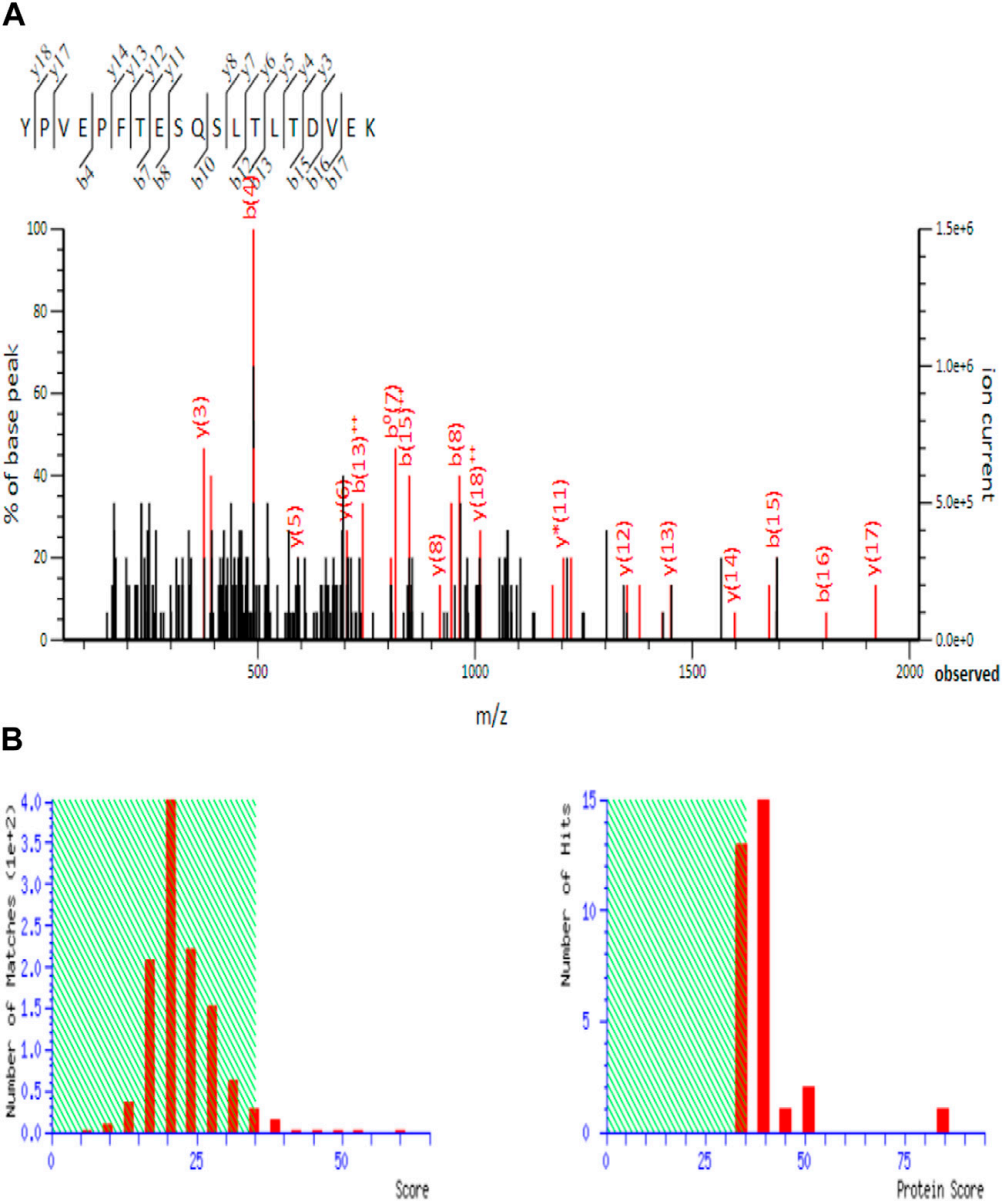
Total ion chromatogram of 2D spot obtained from fermented sheep milk with KGL4 culture (EMS to EPI scan in LC-MS). **(A)** MS/MS spectrum of the peptide sequence inspected in MASCOT database; **(B)** protein score of peptide sequence (found in SwissProt software).

The analysis of 2D spots from the fermentation of sheep milk produced with the KGL4 strain resulted in the detection of 36 spots. Subsequent trypsin digestion and RP-LC/MS study were performed on individual spots for peptide identification. Figure 7 illustrates ion chromatogram with protein score of the whole trypsin-digested 2D spots acquired through the fermentation of sheep milk with the KGL4 culture. Peptide sequences such as ALMGALIK and NMAIHPR from αs2-casein, DMPIQAFLLYQEPVLGPVR and GPFPILV from β-casein, FFSASCVPCVDR from lactotransferrin, LDQWLCE from α-lactalbumin, NMAIHPR through αs2-casein, SCQDQPTAMAR, and SPAQTLQWQVLPNAVPAK from κ-casein, were found based on peptide ranker scores (>0.45) and toxicity (Table 2).

**TABLE 2:** Amino acid sequence obtained from 2D-PAGE of fermented sheep milk with peptide ranking score and physico-chemical properties (*in silico* analysis).

Sequence	Peptide Ranking Score	Prediction	Mol.Wt.	Iso-Electric Point	Net Charge	Hydrophobicity	Hydropathicity	Hydrophilicity
ALMGALIK	0.45043	Non-Toxin	816.19	9.11	1	0.2	1.66	−0.59
DMPIQAFLLYQEPVLGPVR	0.55134	Non-Toxin	2186.89	4.38	−1	0	0.31	−0.39
FFSASCVPCVDR	0.72147	Non-Toxin	1330.68	6.13	0	−0.04	0.8	−0.33
GPFPI LV	0.86929	Non-Toxin	855.21	5.88	0	0.3	1.46	−0.95
LDQWLCE	0.58329	Non-Toxin	906.12	3.67	−2	−0.08	−0.19	−0.26
NMAIHPR	0.46247	Non-Toxin	838.09	10.11	1.5	−0.23	−0.66	−0.13
SCQDQPTAMAR	0.52182	Non-Toxin	1207.48	6.16	0	−0.32	−0.92	0.27
SPAQTLQWQVLPNAVPAK	0.49426	Non-Toxin	1948.52	9.11	1	−0.07	−0.21	−0.43

The peptides DMPIQ*AFLLYQEPVLGP*VR, *GPF*PILV, QAF*LLY*QEPV*LGPV*R, and SCQDQ*PT*AMAR identified in sheep milk fermented with KGL4 exhibited higher peptide ranker scores in Table 3. These sequences have similarities with peptides such as LGP, AF, GP, PT, LLYQEP, YQEPVL, LLYQQPV, AVP (Iwaniak and Dziuba, 2009), AFP, AVPYPQR (Wu et al., 2006), VLGP (Gutiez et al., 2013), GPVGPA (Oshima et al., 1979), LLYQQPV (Gutiez et al., 2013), YQEPVLGPVRGPF (Pihlanto and Makinen, 2013), GPFPILV (Zhang et al., 2016), VR (Gomez-Ruiz et al., 2007), VYPFPG (Wakai and Yamamoto, 2012), YQQPVLGPVR (Meisel and Schlimme, 1990), and FP and AVP (Hernandez-Ledesma and Hsieh, 2013) from the AHTPDB databases. This similarity further confirms their ACE inhibitory activity. In Table 4, the sequences QAFLLYQEP*VLGPVR*, DMPI*QAFLLYQEPVLGPVR*, *GPF*PILV, and HQG*LPQ*EVLNENLLR were identified in sheep milk fermented with KGL4 after trypsin digestion. These sequences partially corresponded to antihypertensive peptide fractions such as QEPVLGPVRGPFP and VR (Gong et al., 2020), RCMAFLLSDGAAAAQQLLPQY (Siow and Gan, 2016), LPQNIPPL and LPQ (Uenishi et al., 2012), GPFPILV (Zhang et al., 2016), VPITPT (Uenishi et al., 2012), and WL (Nongonierma et al., 2014) in the BIOPEP database, providing confirmation of their antidiabetic activity.

**TABLE 3:** Amino acid sequence with antihypertensive activity derived from 2D-PAGE of fermented sheep milk (KGL4) matched with the AHTPDB database.

Sequence	ID	PMID/LINK	Matched sequence	MW	Source	Reference
ALMGALIK	1131	1131_link	DAYPS**GA**W	865.9	Milk	Meisel and Schlimme (1990)
	2274	2274_link	**GA**W	332.36	Milk derived	Wu et al. (2006)
	4385	4385_link	DAYPS**GA**W	865.9	Milk	Kopf-Bolanz. et al. (2014)
	4394	4394_link	DAYPS**GA**	679.68	Casein	Kopf-Bolanz. et al. (2014)
	6957	215231	GPA**GA**P	468.51	Bovine (*Bos taurus*)	Oshima et al. (1979)
	5888	17483275	NIPPLTQTPVVVPPFIQPEV**MG**VSK	2688	Milk (bovine sodium caseinate)	Hayes et al. (2007)
	3379	3379_link	LPQNIPPLTQTPVVVPPFLQPEV**MG**VSK	3026.63	Bovine (*Bos taurus*) β-caseins	Iwaniak and Dziuba (2009)
	4350	23845432	LQPEV**MG**VSK	1086.4	Milk (bovine skim milk)	Gutiez et al. (2013)
DMPIQAFLLYQEPVLGPVR	1183	1183_link	RDMPIQ**AF**	977.15	Milk	Meisel and Schlimme (1990)
	2267	2267_link	**AF**P	333.39	Milk-derived	Wu et al. (2006)
	3419	3419_link	**AF**	236.27	Bovine (*Bos taurus*) β-caseins	Iwaniak and Dziuba (2009)
	4109	4109_link	RDMPIQ**AF**	977.15	Bovine β-Casein	Iwaniak and Dziuba (2009)
	3381	3381_link	LLYQQPVL**GPV**RGPFPIIV	2106.58	Bovine (*Bos taurus*) β-caseins	Iwaniak and Dziuba (2009)
	3382	3382_link	YQQPVL**GPV**R	1156.35	Bovine (*Bos taurus*) β-caseins	Iwaniak and Dziuba (2009)
	3415	3415_link	**GPV**	271.32	Bovine (*Bos taurus*) β-caseins	Iwaniak and Dziuba (2009)
	6961	215231	**GPV**GPA	496.56	Bovine (*Bos taurus*)	Oshima et al. (1979)
	1163	1163_link	LLYQQPV**LGP**VRGPFPIIV	2106.58	Milk	Meisel and Schlimme (1990)
	1216	1216_link	YQQPV**LGP**VR	1156.35	Milk	Meisel and Schlimme (1990)
	3382	3382_link	YQQPV**LGP**VR	1156.35	Bovine (*Bos taurus*) β-caseins	Iwaniak and Dziuba (2009)
	3412	3412_link	**LGP**	285.34	Bovine (*Bos taurus*) β-caseins	Iwaniak and Dziuba (2009)
	3627	3627_link	YQEPV**LGP**VRGPFPIIV	1881.25	Cheese [Cheddar (with probiotics)]	Pihlanto and Makinen (2013)
	3636	3636_link	YQEPV**LGP**VRGPFPI	1668.96	Cheese (Fresco)	Pihlanto and Makinen (2013)
	4355	23845432	V**LGP**	384.2	Milk (bovine skim milk)	Gutiez et al. (2013)
	5553	5553_link	VL**GPV**RGPFP	1038.26	Casein-derived	Baum et al. (2013)
	3381	3381_link	**LLY**QQPVLGPVRGPFPIIV	2106.58	Bovine (*Bos taurus*) β-caseins	Iwaniak and Dziuba (2009)
	3431	3431_link	**LLY**QQPV	860.02	Bovine (*Bos taurus*) β-caseins	Iwaniak and Dziuba (2009)
	4342	23845432	**LLY**QEP	761.3	Milk (Bovine skim milk)	Gutiez et al. (2013)
	3462	3462_link	**YQEPVL**	747.85	Bovine (*Bos taurus*) β-caseins	Iwaniak and Dziuba (2009)
	3627	3627_link	**YQEPVL**GPVRGPFPIIV	1881.25	Cheese [cheddar (with probiotics)]	Pihlanto and Makinen (2013)
	3635	3635_link	**YQEPVL**GPVRGPF	1458.68	Cheese (Fresco)	Pihlanto and Makinen (2013)
	3636	3636_link	**YQEPVL**GPVRGPFPI	1668.96	Cheese (Fresco)	Pihlanto and Makinen (2013)
	5551	5551_link	**YQEPVL**GPVRGPFPIIV	1881.25	Casein-derived	Baum et al. (2013)
	6949	8201050	LL**YQEPVL**GPVRGPFPIIV	2107.57	Bovine (*Bos taurus*)	Yamamoto et al. (1994)
	1002	17948260	**VR**	272.9	Milk (ovine milk proteins)	Gomez-Ruiz et al. (2007)
	1021	24215325	PY**VR**YL	809.96	Milk	Lau et al. (2013)
	1163	1163_link	LLYQQPVLGP**VR**GPFPIIV	2106.58	Milk	Meisel and Schlimme (1990)
	1216	1216_link	YQQPVLGP**VR**	1156.35	Milk	Meisel and Schlimme (1990)
	1338	1338_link	**VR**YL	549.67	Cheese (Manchego)	Hernandez-Ledesma and Hsieh (2013)
	2235	2235_link	**VR**LPT	584.72	Milk	Gu et al. (2011)
	2236	2236_link	**VR**PEK	627.74	Milk	Gu et al. (2011)
	2614	24915368	PY**VR**YL	809.96	Milk (Caprine Kefir)	Puchalska et al. (2015)
	3545	3545_link	**VR**	273.34	Bovine lactoferrin (*Bos taurus*)	Iwaniak and Dziuba (2009)
	3627	3627_link	YQEPVLGP**VR**GPFPIIV	1881.25	Cheese (cheddar (with probiotics))	Pihlanto and Makinen (2013)
	3380	3380_link	R**DM**PIQAF	977.15	Bovine (*Bos taurus*) b-caseins	Iwaniak and Dziuba (2009)
	3672	21779574	YPQR**DM**PIQ	1147.31	Casein	Phelan and Kerins (2011)
	4109	4109_link	R**DM**PIQAF	977.15	Bovine β-casein	Iwaniak and Dziuba (2009)
	4360	23845432	PQR**DM**P	742.3	Milk (bovine skim milk)	Gutiez et al. (2013)
	6076	15537298	QKAVPYPQR**DM**PI	1543	Milk (sodium caseinate)	Robert et al. (2004)
FFSASCVPCVDR	1657	1657_link	A**VP**YPQR	829.95	Casein	Wu et al. (2006)
	2249	2249_link	A**VP**	285.34	Milk-derived	Iwaniak and Dziuba (2009)
	3383	3383_link	A**VP**	285.34	Bovine (*Bos taurus*) β-caseins	Iwaniak and Dziuba (2009)
	3393	3393_link	A**VP**YPQR	829.95	Bovine (*Bos taurus*) β-caseins	Iwaniak and Dziuba (2009)
	4065	4065_link	A**VP**	285.34	Bovine β-caseins	Hernandez-Ledesma and Hsieh (2013)
	1189	1189_link	TPVV**VP**PFLQP	1193.45	Milk	Puchalska et al. (2015)
	1165	1165_link	LPQNIPPLTQTPVV**VP**PFLQP EVMGVSK	3026.63	Milk	Dziuba et al. (2009)
	1347	1347_link	**FF**VAPFPEVFGK	1384.64	β-caseins	Hernandez-Ledesma and Hsieh (2013)
	1656	1656_link	**FF**VAPFPEVFGK	1384.64	Casein	Wakai and Yamamoto (2012)
	2010	2010_link	**FF**VAPFEVFGK	1287.52	Casein hydrolysate	Gu et al. (2011)
	2948	2948_link	**FF**VAPFPQVFGF	1402.66	Casein	Aluko (2015)
	2948	2948_link	**FF**VAPFPQVFGF	1402.66	Casein	Aluko (2015)
	4142	22249830	**FF**VAPFPGVFGK	1312.58	Milk proteins	Martinez-Maqueda et al. (2012)
GPFPI LV	1334	1334_link	**FP**	262.32	Bovine serum albumin	Hernandez-Ledesma and Hsieh (2013)
	1347	1347_link	FFVA**PFP**EVFGK	1384.64	β-Caseins	Hernandez-Ledesma and Hsieh (2013)
	1707	21185549	LVY**PFP**GPIPN	1213.44	Milk (Kefir from caprine milk)	Hernandez-Ledesma et al. (2011)
	1163	1163_link	LLYQQPVLGPVRG**PFP**IIV	2106.58	Milk	Meisel and Schlimme (1990)
	1707	21185549	LVY**PFP**GPIPN	1213.44	Milk (Kefir from caprine milk)	Hernandez-Ledesma et al. (2011)
	1709	21185549	Y**PFP**GPIPN	1001.15	Cheese (gouda)	Hernandez-Ledesma et al. (2011)
	1347	1347_link	FFVA**PFP**EVFGK	1384.64	β-caseins	Hernandez-Ledesma et al. (2013)
	1662	1662_link	VY**PFP**G	678.79	Casein	Wakai and Yamamoto (2012)
	1709	21185549	Y**PFP**GPIPN	1001.15	Cheese (Gouda)	Hernandez-Ledesma et al. (2011)
	6957	215231	**GP**AGAP	468.51	Milk	Oshima et al. (1979)
	6961	215231	**GP**VGPA	496.56	Bovine (*Bos taurus*)	Oshima et al. (1979)
	4626	4626_link	NA**GP**FTPTVNREQLSTS	1818.96	Ewes raw milk cheese	Sagardia et al. (2013)
LDQWLCE	1305	1305_link	**WL**AHK	653.78	Whey (whole whey protein)	Hernandez-Ledesma and Hsieh (2013)
	1332	1332_link	**WL**AHK	653.78	Alpha-lactoglobulin	Tavares and Malcata (2013)
	3342	3342_link	VGINY**WL**AHK	1200.41	Bovine b-casein	Iwaniak and Dziuba (2009)
	3371	3371_link	**TQ**SLVYP	806.91	Bovine (*Bos taurus*) β-caseins	Iwaniak and Dziuba (2009)
NMAIHPR	1670	1670_link	IY**PR**Y	710.83	Casein	Wakai and Yamamoto (2012)
	4131	4131_link	Y**PR**	434.5	Bovine β-lactoglobulin	Iwaniak and Dziuba (2009)
	5703	5703_link	TQP**KT**NAIPY	1132	Cheese (Manchego cheese)	Gomez-Ruiz et al. (2007)
	4324	4324_link	M**AI**PPK	655.85	Milk-Cheese (ewes milk and cheese proteins)	Hernandez-Ledesma and Hsieh (2013)
	5703	5703_link	TQPKTN**AI**PY	1132	Cheese (Manchego cheese)	Gomez-Ruiz et al. (2007)
QAFLLYQEPV LGPVR	1216	1216_link	YQQPV**LGP**VR	1156.35	Milk	Meisel and Schlimme (1990)
	3381	3381_link	LLYQQPV**LGP**VRGPFPIIV	2106.58	Bovine (*Bos taurus*) β-caseins	Iwaniak and Dziuba (2009)
	1216	1216_link	YQQPVL**GPV**R	1156.35	Milk	Meisel and Schlimme (1990)
	3382	3382_link	YQQPVL**GPV**R	1156.35	Bovine (*Bos taurus*) β-caseins	Iwaniak and Dziuba (2009)
	3381	3381_link	**LLYQ**QPVLGPVRGPFPIIV	2106.58	Bovine (*Bos taurus*) β-caseins	Iwaniak and Dziuba (2009)
	6949	8201050	**LLY**QEPVLGPVRGPFPIIV	2107.57	Bovine (*Bos taurus*)	Yamamoto et al. (1994)
	4089	4089_link	**LLY**QQPVLGPVRGPFPIIV	2106.58	Bovine β-casein	Iwaniak and Dziuba (2009)
SCQDQPTAMAR	1333	1333_link	ALKAWSV**AR**	1001.2	Bovine serum albumin	Tavares and Malcata (2013)
	1790	1790_link	**AR**HPHPHLSF	1198.35	Casein	Del Mar Contreras et al. (2009)
	2189	2189_link	**AR**HEI	624.7	Milk	Gu et al. (2011)
	4267	21773582	ALKAWSV**AR**	1001.2	Bovine whey proteins	Nagpal et al. (2011)
	2089	2089_link	**AR**Y	408.46	Milk-derived	Gu et al. (2011)
	2092	2092_link	GGR**PT**Y	649.7	Milk-derived	Gu et al. (2011)
	2285	2285_link	I**PT**	329.4	Milk-derived	Del Mar Contreras et al. (2009)
	3443	3443_link	**PT**	216.24	Bovine (*Bos taurus*) β-lactoglobulin -caseins	Iwaniak and Dziuba (2009)
	1189	1189_link	TPVVVPPFL**QP**	1193.45	Milk	Puchalska et al. (2015)
	1791	1791_link	QSWMHQPH**QP**LPPTVM	1914.23	Casein	Del Mar Contreras et al. (2009)
	2288	2288_link	I**QP**	356.42	Milk derived	Wu et al. (2006)
SPAQTLQWQVLPNAVPAK	1657	1657_link	**AVP**YPQR	829.95	Casein	Wu et al. (2006)
	2249	2249_link	**AVP**	285.34	Milk derived	Iwaniak and Dziuba (2009)
	3383	3383_link	**AVP**	285.34	Bovine (*Bos taurus*) β-caseins	Iwaniak and Dziuba (2009)
	3393	3393_link	**AVP**YPQR	829.95	Bovine (*Bos taurus*) β-caseins	Iwaniak and Dziuba (2009)
	4065	4065_link	**AVP**	285.34	Bovine β-caseins	Hernandez-Ledesma and Hsieh (2013)
	1313	1313_link	**LQ**KW	573.69	Whey protein (β-lactoglobulin)	Del Mar Contreras et al. (2009)
	1802	1802_link	PVVVPPF**LQ**	995.23	Casein	Del Mar Contreras et al. (2009)
	2012	2012_link	**LQ**KW	573.69	β-lactoglobulin	Dziuba et al. (2009)
	1167	1167_link	**LQ**SW	532.6	Milk	Hernandez-Ledesma and Hsieh (2013)

**TABLE 4:** Amino acid sequence with antidiabetic activity derived from 2D-PAGE of fermented sheep milk (KGL4) matched with the BIOPEP database.

Sequence	ID	Matched Sequence	MW	Source	Reference
ALMGALIK	8627	IPAVFKID**AL**	1086.3203	α-Lactalbumin	Lacroix and Li-Chan (2014)
	8665	KVSVV**AL**	714.8911	Cowpea bean	De Souza Rocha et al. (2014)
	8666	VKSVV**AL**	714.8911	Cowpea bean	De Souza Rocha et al. (2014)
	10292	PPH**MG**GP	691.7981	Pinto bean	Ngoh and Gan (2018)
	9802	GQLGEHGGAG**MG**	1070.1357	Pinto bean	Złotek et al. (2020)
	10386	MA**MG**	408.5377	Wheat gluten protein	Ding et al. (2022)
	10388	MA**MG**L	521.6949	Wheat gluten protein	Ding et al. (2022)
DMPIQAFLLYQEPVLGPVR	8593	**VLGP**	384.462	Casein	Nongonierma and FitzGerald (2013)
	9686	**QEPVLGPVR**GPFP	1392.61	Goat milk β-casein	Gong et al. (2020)
	8664	TATG**LL**E	703.777	Cowpea bean	De Souza Rocha et al. (2014)
	8726	LRENNKLM**LL**ELK	1613.97	Common bean (*Phaseolus vulgaris)*	Mojica et al. (2015)
	8732	RL**LL**KLRQ	1039.32	Common Bean (*Phaseolus vulgaris*)	Mojica et al. (2015)
	9686	QEPVLGP**VR**GPFP	1392.61	Goat milk β-casein	Gong et al. (2020)
	10309	CGKKF**VR**	837.0432	Dark tea protein	Zhao et al. (2020)
	9686	QEPVLGP**VR**GPFP	1392.61	Goat milk β-casein	Gong et al. (2020)
	9409	VPPF**IQ**PE	926.068	Gouda cheese	Uenishi et al. (2012)
	8552	RNDDLNY**IQ**	1150.2	Egg-yolk protein	Zambrowicz et al. (2015)
	8735	LSERRMLLRKEK**QA**Q	1886.24	Common Bean (*Phaseolus vulgaris*)	Mojica et al. (2015)
FFSASCVPCVDR	9409	**VP**PFIQPE	926.068	Gouda cheese	Uenishi et al. (2012)
	8658	**VP**ITPT	626.737	Gouda-type cheese	Uenishi et al. (2012)
	9023	IA**VP**TGVA	726.858	Soy and lupin protein	Lammi et al. (2016)
	9249	**VP**ITPTL	739.897	Gouda-type cheese	Uenishi et al. (2012)
	9023	IA**VP**TGVA	726.858	Soy and lupin protein	Lammi et al. (2016)
	10214	**VP**LVM	557.741	Broccoli	Pei et al. (2022)
	9556	**VP**YPQ	602.668	Casein	Zheng et al. (2019)
	9389	R**VP**SLM	701.876	Egg white protein	Yu et al. (2011)
	9393	R**VP**SL	570.678	Egg white protein	Yu et al. (2011)
SCQDQPTAMAR	10300	DPA**QP**NYPWTAVLVFRH	2011.2361	Cumin seeds	Siow and Gan (2016)
	8589	LK**PT**PEGDLEIL	1324.5150	α-Lactalbumin	Lacroix and Li-Chan (2014)
	8663	VPIT**PT**	626.7401	Casein	Uenishi et al. (2012)
	8863	L**PT**EV	557.6353	Cowpea bean	De Souza Rocha et al. (2014)
	10303	IAV**PT**GVA	726.8589	Soy and lupin protein	Lammi et al., 2016
SPAQTLQWQVLPNAVPAK	8839	NI**NA**HSVVY	1016.1053	Oat bran	Esfandi et al. (2022)
	9202	DPAQ**PN**YPWTAVLVFRH	2011.25	Cumin seeds	Siow and Gan (2016)
	10115	YPFPGPI**PN**	1001.13	Gouda cheese	Uenishi et al. (2012)
	10300	DPAQ**PN**YPWTAVLVFRH	2011.2361	Cumin seeds	Siow and Gan (2016)
GPFPI LV	8662	VDT**FP**A	648.7	Cowpea bean	De Souza Rocha et al. (2014)
	8668	DVT**FP**A	648.7	Cowpea bean	De Souza Rocha et al. (2014)
	8669	EVT**FP**A	662.727	Cowpea bean	De Souza Rocha et al. (2014)
	TD	DLT**FP**A	662.727	Cowpea bean	De Souza Rocha et al. (2014)
	9024	LT**FP**GSAED	935.975	Soy and lupin protein	Lammi et al. (2016)
	9128	GP**FP**ILV	741.916	Bovine and caprine milk casein	Zhang et al. (2016)
	9408	**FP**GPIPD	741.829	Gouda cheese	Uenishi et al. (2012)
	9410	YP**FP**GPIPD	1002.12	Gouda cheese	Uenishi et al. (2012)
	9686	QEPVLGPVRGP**FP**	1392.61	Goat milk casein	Gong et al. (2020)
	9128	GPFPI**LV**	741.916	Bovine and caprine milk casein	Zhang et al. (2016)
	10214	VP**LV**M	557.741	Broccoli	Pei et al. (2022)
	10213	VP**LV**M	557.741	Broccoli	Pei et al. (2022)
	10214	VP**LV**M	557.741	Broccoli	Pei et al. (2022)
	9410	Y**PF**PGPIPD	1002.12	Gouda cheese	Uenishi et al. (2012)
	9931	G**PF**PLLV	741.916	Tilapia byproduct hydrolysate	Theysgeur et al. (2020)
	9409	VP**PF**IQPE	926.068	Gouda cheese	Uenishi et al. (2012)
	10115	Y**PF**PGPIPN	1001.13	Gouda cheese	Uenishi et al. (2012)
	9408	FPG**PI**PD	741.829	Gouda cheese	Uenishi et al. (2012)
	9410	YPFPG**PI**PD	1002.12	Gouda cheese	Uenishi et al. (2012)
	10115	YPFPG**PI**PN	1001.13	Gouda cheese	Uenishi et al. (2012)
	9683	SDIPN**PI**GSE	1028.08	Goat milk casein	Gong et al. (2020)
	9128	GPF**PI**LV	741.916	Bovine and caprine milk casein	Zhang et al. (2016)
	9249	V**PI**TPTL	739.897	Gouda-type cheese	Uenishi et al. (2012)
	8658	V**PI**TPT	626.737	Gouda-type cheese	Uenishi et al. (2012)
	9128	**GPF**PILV	741.916	Bovine and caprine milk casein	Zhang et al. (2016)
	9686	QEPVLGPVR**GPF**P	1392.61	Goat milk casein	Gong et al. (2020)
	9686	QEPVLGPVR**GPF**P	1392.61	Goat milk β-casein	Gong et al. (2020)
	9202	DPAQPNYPWTA**VL**VFRH	2011.2361	casein-derived peptide	Siow and Gan (2016)
	8593	**VL**GP	384.4692	β-lactoglobulin	Nongonierma and FitzGerald (2013)
	8922	**VL**	230.3031	Egg white protein	Lan et al. (2015)
	9405	N**VL**QPS	656.7264	Egg white protein	Yu et al. (2011)
	9417	NVLQPS	656.7264	α-Lactalbumin	Yu et al. (2012)
LDQWLCE	8626	LCSEKLDQ**WL**	1234.4188	Milk protein	Lacroix and Li-Chan (2014)
	8677	**WL**	317.3819	Soy protein	Nongonierma et al. (2014)
	8625	WCKD**DQ**NPHS	1229.2770	α-Lactalbumin	Lacroix and Li-Chan (2014)
	8626	LCSEKL**DQ**WL	1234.4188	α-Lactalbumin	Lacroix and Li-Chan (2014)
	8635	CKD**DQ**NPHSS	1130.1446	Goat milk casein	Lacroix and Li-Chan (2014)
	9684	NPW**DQ**VKR	1042.1458	α-Lactalbumin	Gong et al. (2020)
	8626	LCSEKLD**QWL**	1234.4188	Cumin seeds (*Cuminum cyminum*)	Lacroix and Li-Chan (2014)
NMAIHPR	9200	RC**MA**FLLSDGAAAAQQLLPQYW	2453.8313	Wheat gluten protein	Siow and Gan (2016)
	10386	**MA**MG	408.5377	Wheat gluten protein	Ding et al. (2022)
	10388	**MA**MGL	521.6949	Casein	Ding et al. (2022)
	9127	**HP**INHR	772.8529	Wheat germ	Zhang et al. (2015)
	10306	SQHISTAGMEASGTS**NM**KF	1984.1687	Cumin seeds (*Cuminum cyminum*)	Liu et al. (2021)
QAFLLYQEPV LGPVR	9200	RCMA**FL**LSDGAAAAQQLLPQYW	2453.8313	Cumin seeds (*Cuminum cyminum*)	Siow and Gan (2016)
	8591	**FL**QP	503.585	Casein	Nongonierma and FitzGerald (2013)
	9686	QEPVLGP**VR**GPFP	1392.61	Goat milk β-casein	Gong et al. (2020)
	9200	RCM**AFLL**SDGAAAAQQLLPQYW	2453.8313	Wheat gluten protein	Siow and Gan (2016)
	10299	HNKPEVE**VR**	1107.2173	Egg	Qiu et al. (2020)
	10309	CGKKF**VR**	837.0432	Dark tea protein	Zhao et al. (2020)

### 3.8 The inhibitory effects of fermented sheep milk on RAW macrophage cells

#### 3.8.1 Cell viability

**FIGURE 8:**
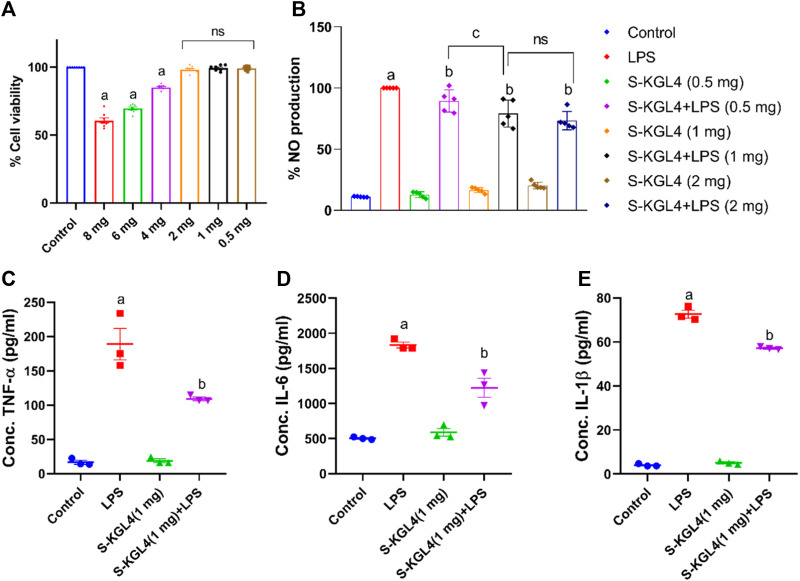
Effect of S-KGL4 on **(A)** cell viability (MTT assay) of RAW 264.7 cells, **(B)** NO production in S-KGL4, **(C)** TNF-α, **(D)** IL-6, **(E)** IL-1β measured in the supernatants of LPS-stimulated RAW 264.7 Cell data are presented as mean ± SEM; *n* = 3 and evaluated by one-way ANOVA followed by Tukey’s *post hoc* test. * relative to the control, # relative to the LPS, LPS: lipopolysaccharide. *p*-value for # is 0.0124, 0.0069, and <0.0001 in TNF-α, IL-6, and IL-1β, respectively.

The 264.7 RAW cells underwent MTT analysis to evaluate cell viability after being treated with sheep milk fermented using the *Lactobacillus* culture, *Limosilactobacillus fermentum* (KGL4). The experiment comprised six different concentrations of fermented sheep milk (0.5, 1, 2, 4, 6, and 8 mg/mL) along with a control group (without cultures). The findings depicted in Figure 8A reveal a decline in cell viability rising from 0.5 to 8 mg/mL in concentration. The KGL4 strain especially demonstrated the non-cytotoxic effects from 0.5, 1, and 2 mg/mL concentrations, resulting in nearly 100% maximum viability of cells. However, at higher concentrations (4, 6, and 8 mg/mL), cytotoxicity escalated, leading to a reduction in the viability of cells.

#### 3.8.2 Impact of cultured sheep milk upon pro-inflammatory cytokine and nitric oxide (NO) percentages in RAW 264.7 cells

Considerable production in inflammatory mediator nitric oxide occurs in RAW 264.7 cells induced with LPS. TNF-α, IL-6, and IL-1β are key contributors to the reaction of inflammation and the beginning of inflammation and are classified as pro-inflammatory cytokines. This research explores the influence of different concentrations of S-KGL4-fermented sheep milk on 264.7 RAW cells, assessing both the nitric oxide percentage and its impact on pro-inflammatory cytokines. As depicted in Figure 8B, there was a significant rise in nitrite levels observed in the LPS group, but this increase was effectively alleviated in the group receiving fermented sheep milk along with LPS treatment. The group treated with only fermented sheep milk exhibited the lowest nitrite production, nearly comparable to the control group. This finding implies that the generation of peptides via fermented sheep milk does not trigger any harmful effects on 264.7 RAW cells. The results presented in Figures 8C–E illustrate the impact of fermented sheep milk at a 1 mg/mL concentration on TNF-α, IL-6, and IL-1β pro-inflammatory cytokines. Investigation showed a noteworthy decline in the excessive manufacturing of TNF-α, IL-6, and IL-1β triggered via activation with LPS when using KGL4-fermented sheep milk. The values for S-KGL4-fermented sheep milk closely resembled those of the control, suggesting a noteworthy reduction in pro-inflammatory cytokine levels. In Khakhariya et al. (2023), 264.7 RAW cells underwent 24-h of incubation. Fully matured macrophages were exposed to lipopolysaccharide (LPS) at a concentration of 1 g/mL in a combination of *Lacticaseibacillus paracasei* (M11, MG027695) and *Saccharomyces cerevisiae* (WBS2A, MG101828). These bacteria were fermented with, respectively, buffalo and camel milk. Following this, the cells were cultured about 16 h in a CO_2_ incubator with controlled humidity. The study findings highlight the significance of the anti-inflammatory response in the context of inflammatory diseases. Camel and buffalo milk fermented with M11 and WBS2A shows promise as a potent source of anti-inflammatory peptides, demonstrated by a decrease in the production of cytokines associated with inflammation. These results indicate that the fermented sheep milk’s inflammatory properties were reduced due to the generation of bioactive compounds such as short-chain fatty acids and bioactive peptides during the fermentation process. Sansi et al. (2023) investigated the potential anti-inflammatory attributes of peptides obtained from CPH (casein protein hydrolysates) and WPH (whey protein hydrolysates) in combination with lipopolysaccharides (LPS). Within the dosage range of 50–550 g/mL, both CPH and WPH peptide fractions demonstrated no harmful effects on the cell line HT-29. The viability of the cells remained high for both types of peptides, showing survival rates of up to 80%, and no discernible statistically significant variations were noted. However, our outcomes indicate that the WPH and CPH peptide fractions exhibited moderate cytotoxicity while maintaining substantial cell selectivity in our study. Additionally, our examination of fermented sheep milk revealed a more potent anti-inflammatory impact on cells aimed at mitigating excessive production of inflammatory cytokines and mediators. Importantly, KGL4 did not exhibit any inflammatory effects in our assessment.

### 3.9 CLSM (confocal laser scanning microscopy) and particle size of fermented sheep milk

**FIGURE 9:**
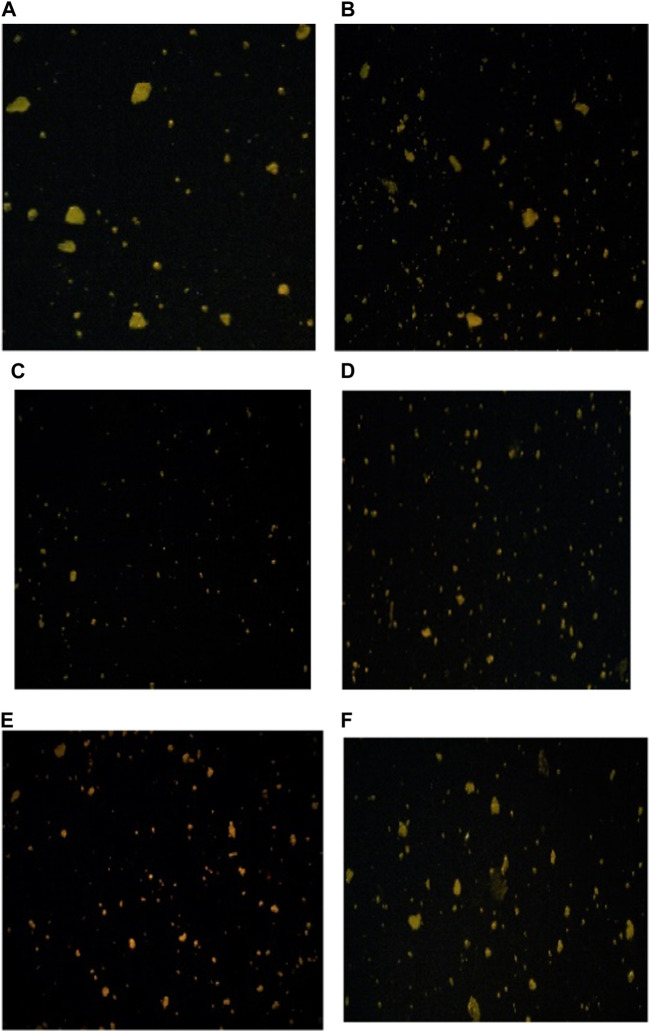
CLSM images showing the microstructure of fermented sheep milk using KGL4 strain. **(A)** Unfermented sheep milk sample, **(B)** fermented sheep milk sample, **(C)** <3 kDa permeate sample, **(D)** >3 kDa retentate sample, **(E)** <10 kDa permeate sample, and **(F)** >10 kDa retentate sample.

**FIGURE 10:**
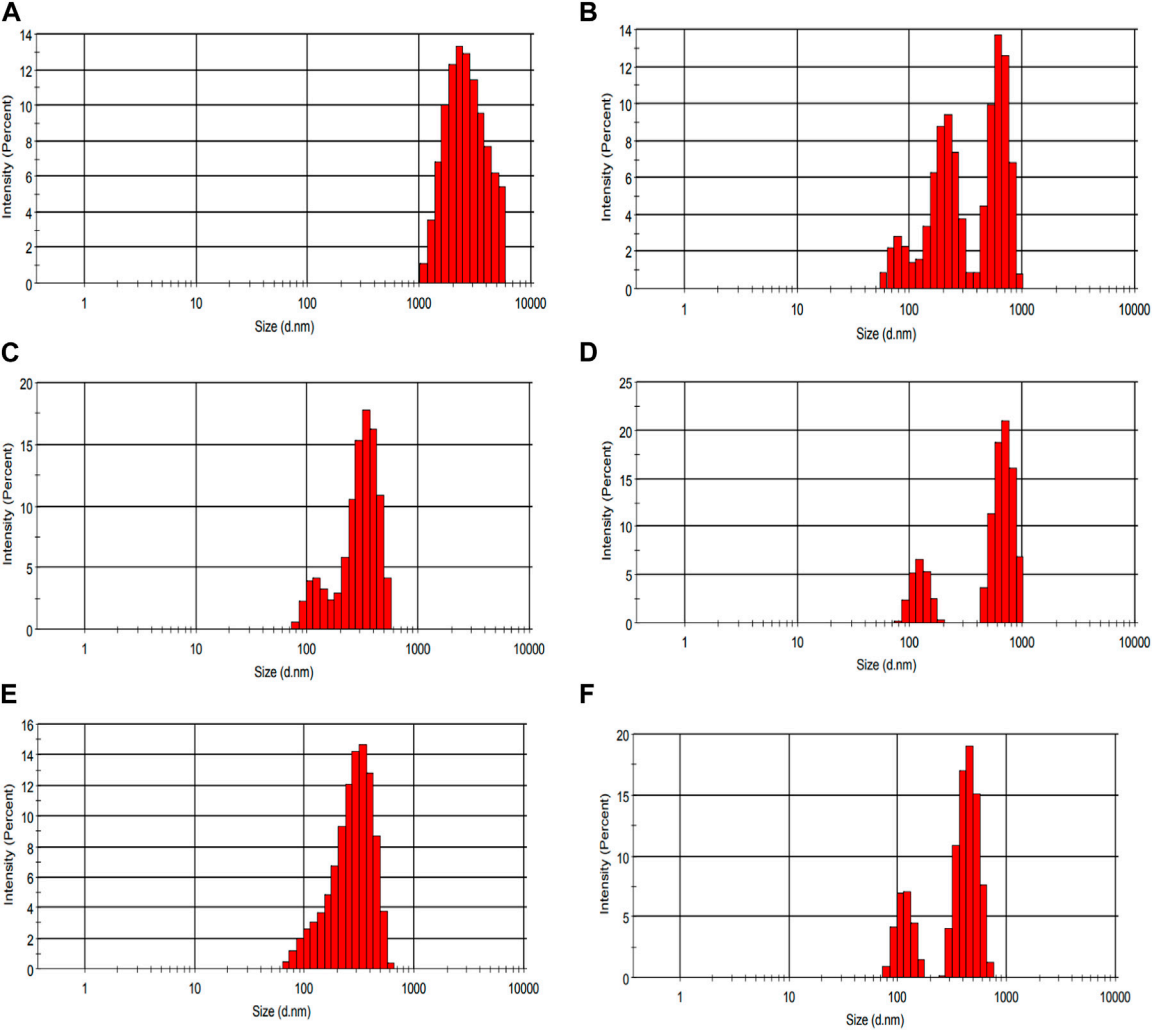
Particle size distribution of the waster-soluble extract of fermented sheep milk using KGL4 strain. **(A)** Unfermented sheep milk sample size, **(B)** fermented sheep milk sample size, **(C)** <3 kDa permeate sample size, **(D)** >3 kDa retentate sample size, **(E)** <10 kDa permeate sample size, **(F)** >10 kDa retentate sample size.

Figure 9 demonstrates the use of CLSM (confocal laser scanning microscopy) to examine the micro-organization of unfermented sheep milk (UFM), specifically focusing on aspects such as the protein network, peptide binding, and overall composition. When analyzing KGL4 strain-produced water-soluble extracts using fermented sheep’s milk (FSM) and its fractions, the application of fluorescein dye highlighted proteins in a green coloration. Notably, the UFM sample reveals the existence of larger particles, indicative of a native protein structure, in contrast to FSM, where proteins undergo degradation into peptides during the fermentation process. Additionally, FSM exhibits more intricate protein structures and aggregated larger protein formations. This phenomenon is attributed to the unique capability of the KGL4 strain of the *Lactobacillus* culture to convert proteins into diverse polymers throughout the fermentation process. The permeate and retentate specimens derived from FSM subjected to membrane treatment demonstrate molecular weight cut-offs of 3 and 10 kDa. The identification of smaller protein structures in the 3 kDa permeate sample implies the existence of diminutive peptides in the FSM sample following treatment with the 3 kDa membrane, in contrast to the 3 kDa retentate, 10 kDa permeate, and 10 kDa retentate models. No substantial distinctions were noted in FSM samples with molecular weights exceeding 3 kDa but less than 10 kDa. Nevertheless, the 10 kDa permeate sample exhibits larger particles than samples treated with other cut-off membranes. In Figure 10, it is clear that unfermented sheep milk (USM) exhibited greater particle dimensions than fermented sheep milk (FSM). This difference is attributed to the strong proteolytic activity of KGL4. As a result, smaller particles were observed in the 3 and 10 KDa permeates of FSM, contrasting with the 3 and 10 KDa retentates after the fermentation process. Ningtyas et al. (2021) studied the effect of fermentation in soymilk and yogurt through CLSM and particle size analysis. Fermented yogurt culture undergoes protein coagulation due to lactic acid production and enzymatic activity, forming a coagulated mass and significant observable changes in protein structures through CLSM. The fermented dairy protein network formed a looser protein network with a higher number of pores (Boeck et al., 2021). The microstructural analysis of yogurt using a different culture for fermentation showed significant differences between samples fermented by different cultures. These findings emphasize the influence of fermentation conditions and microbial strains on the final product’s microstructure in CLSM images (Nguyen et al., 2023).

### 3.10 Molecular interaction study of peptides from fermented sheep milk

Over the last 10 years, molecular docking has become an essential instrument for examining the structural relationships between various ligands, including bioactive peptides, with specific enzyme active sites. This makes it possible for researchers to analyze and enhance the molecular connections between the objective proteins and examine peptide ligands (Singh et al., 2024). With this research, peptides were recognized through 2D gel and RP-LC/MS techniques, and *in silico* approaches were employed to investigate their interactions with particular enzymes. The peptide GPFPILV was selected for docking with pancreatic α-amylase and angiotensin-converting enzyme (ACE) based on its highest peptide ranking score and other relevant properties (Table 2). Considering the role of α-amylase in breaking down starch and glycogen for efficient absorption during digestion, it is reasonable to anticipate potential antidiabetic properties for the identified peptides using α-amylase (Mudgil et al., 2023b). Conversely, the possible antihypertensive effect of the discovered peptide GPFPILV was predicted using ACE. Within the angiotensin–renin system, ACE performs a crucial role in transforming angiotensin I to active angiotensin II vasoconstrictor, leading to blood vessel constriction and indirectly raising blood pressure (Singh et al., 2014). The RCSB-Protein Data Bank was utilized to obtain the 3D structures of the targeted enzymes, while the Active Site Prediction server was employed to predict their active sites. The peptide GPFPILV was 3D-structured using the Colab server according to the method outlined by Singh, Paul, and Goel in 2024. Docking experiments involving α-amylase and ACE enzymes were conducted using HPEPDOCK v2.0 and AutoDock Vina v1.2.0. The resultant complexes revealed diverse interactions between molecules, such as hydrophobic interactions and hydrogen bonding. As per LigPlot+ analysis conducted with AutoDock Vina, it was predicted that GPFPILV could form up to four hydrogen bonds with α-amylase, specifically interacting with residues Lys261, Lys13, Thr264, and Gly20. Additionally, the analysis indicated potential hydrophobic interactions with residues Leu18, Glu272, Ser270, Thr23, Ala22, Trp284, Lys257, Gly238, Leu237, and Gln19. Concerning ACE, the analysis suggested a maximum of three hydrogen bonds with residues Tyr135, Arg124, and Ser517, along with hydrophobic interactions involving residues Ile204, Glu123, Tyr62, Ser516, Asn85, Ile88, Trp59, Tyr360, Asn66, Leu139, Lpr702, and Trp220 (Supplementary Figure S1). These findings imply that GPFPILV might impede the active and binding sites of the enzymes, potentially hindering substrate binding and affecting their overall functionality. The robustness of protein–peptide complexes is ascribed to robust hydrogen bonds, a formidable intermolecular force. Furthermore, the affinity for binding the ligand within the connection pockets of the intended proteins is influenced by hydrophobic interactions (Khakhariya et al., 2023; Singh et al., 2024). Regarding binding affinity, GPFPILV demonstrated the most elevated values, registering −6.6 kcal/mol and −9.9 kcal/mol for α-amylase and ACE, respectively (Supplementary Table S1). The new HPEPDOCK web server intended for blind protein–peptide docking employs an algorithm with hierarchies, which eliminates the need for extensive modeling to improve the conformations of peptides. Instead, it integrates peptide pliability by utilizing a collection of conformations produced by the MODPEP *de novo* peptide modeling technique. In the investigation of potential binding mechanisms with specific enzymes, the peptide was subjected to docking analysis on the HPEPDOCK server (Mudgil et al., 2023b). Notably, the peptide GPFPILV demonstrated the highest HPEPDOCK scores of −171.717 and −200.641 against α-amylase and ACE, respectively. The comparison of findings across studies is challenging due to the varied scoring algorithms and patterns employed by different molecular docking tools. Nonetheless, the results of molecular docking analysis suggest that the peptides identified by interactions with various residues surrounding and within the active site are able to effectively limit the activities of the specific enzymes, principally through hydrogen bonding and hydrophobic interactions. Using this peptide structure, molecular docking is performed using Auto Dock Vina and HADDOCK software. The reason behind this is to validate the presence of hydrogen bonds and strong bonding. Further studies on dynamic simulations are required to assess the thermodynamic and dynamic properties.

## 4 Conclusion

The application of the *Limosilactobacillus fermentum* KGL4 (MF951099) culture in the fermentation of sheep milk demonstrated significant effects, including ACE inhibition, antidiabetic properties (such as inhibiting α-glucosidase, α-amylase, and lipase), and proteolytic activity. The highest levels of ACE inhibition (74.82%), α-amylase (70.02%), α-glucosidase (72.19%), and lipase inhibition (67.08%) were observed following a 48-h fermentation period, particularly when using a 2.5% inoculation rate (peptide: 9.88 mg/mL). The evaluation of proteolytic activity using OPA peaked after 48 h of incubation. The membrane fractions with an ultrafiltration cutoff showed a peak distribution predominantly between 3 and 10 kDa. Fermented sheep milk demonstrated notable inhibitory effects on ACE (76.69%), α-amylase (75.69%), α-glucosidase (66.27%), and lipase (71.67%). In experiments involving RAW 264.7 cells, S-KGL4 significantly attenuated excessive production of LPS activation inducing TNF-α, IL-6, and IL-1β which suggested potential anti-inflammatory properties. SDS-PAGE analysis detected protein fractions of 51–70 kDa and 10–32 kDa in fermented sheep milk, whereas 2D gel electrophoresis revealed 36 spots corresponding to the KGL4 strain with a molecular weight of 10–32 kDa. Furthermore, it was indicated that peptides with lower molecular weights demonstrate increased potential for ACE inhibition and antidiabetic effects. The study’s general inference is that fermented sheep milk containing *Lactobacillus* is a potentially valuable reservoir of peptides with antihypertensive and antidiabetic properties. However, it is crucial to conduct additional clinical studies to validate these health-related claims.

## Data Availability

The original contributions presented in the study are included in the article/Supplementary Material; further inquiries can be directed to the corresponding author.
