# Supplementary material for: Peptidomics-based identification of antihypertensive and antidiabetic peptides from sheep milk fermented using Limosilactobacillus fermentum KGL4 MTCC 25515 with anti-inflammatory activity: in silico, in vitro, and molecular docking studies
Source: Front Chem. 2024 Apr 30;12:1389846. doi: 10.3389/fchem.2024.1389846 (PMC11091447; doi:10.3389/fchem.2024.1389846)
Supplement: Supplementary file 1 [file DataSheet1.docx]

SUPPLEMENTARY TABLE 1

Molecular docking results of peptide “GPFPILV” identified in fermented sheep milk with targeted enzymes.

|  | **Autodock Vina Binding energy Binding affinity (kcal/mol)** | **Hydrogen bonds** | **HPEPDOCK score** |
| --- | --- | --- | --- |
| **Angiotensin-converting enzyme** | -9.1 | 3 | -200.641 |
| **α-amylase** | -6.6 | 4 | -171.717 |


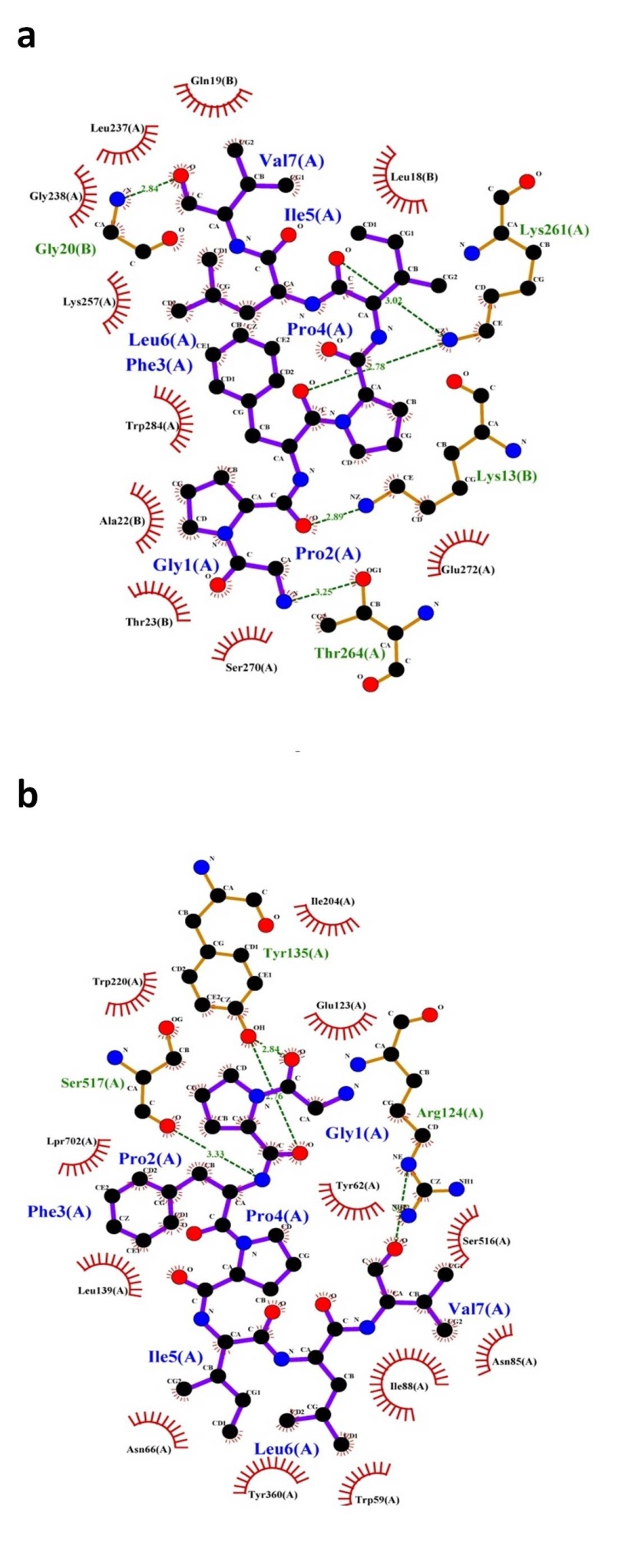


Supplementary Figure 1 2D representation of receptor-ligand interaction of peptide “GPFPILV” with the binding sites of target receptors. GPFPILV- α-amylase [a] and GPFPILV-ACE [b].
